# Distribution of the Age of Gossip in Networks

**DOI:** 10.3390/e25020364

**Published:** 2023-02-16

**Authors:** Mohamed A. Abd-Elmagid, Harpreet S. Dhillon

**Affiliations:** Wireless@VT, Bradley Department of Electrical and Computer Engineering, Virginia Tech, Blacksburg, VA 24061, USA

**Keywords:** Age of Information, information freshness, gossip networks, stochastic hybrid systems

## Abstract

We study a general setting of gossip networks in which a source node forwards its measurements (in the form of status updates) about some observed physical process to a set of monitoring nodes according to independent Poisson processes. Furthermore, each monitoring node sends status updates about its information status (about the process observed by the source) to the other monitoring nodes according to independent Poisson processes. We quantify the freshness of the information available at each monitoring node in terms of Age of Information (AoI). While this setting has been analyzed in a handful of prior works, the focus has been on characterizing the average (i.e., marginal first moment) of each age process. In contrast, we aim to develop methods that allow the characterization of higher-order marginal or joint moments of the age processes in this setting. In particular, we first use the stochastic hybrid system (SHS) framework to develop methods that allow the characterization of the stationary marginal and joint moment generating functions (MGFs) of age processes in the network. These methods are then applied to derive the stationary marginal and joint MGFs in three different topologies of gossip networks, with which we derive closed-form expressions for marginal or joint high-order statistics of age processes, such as the variance of each age process and the correlation coefficients between all possible pairwise combinations of age processes. Our analytical results demonstrate the importance of incorporating the higher-order moments of age processes in the implementation and optimization of age-aware gossip networks rather than just relying on their average values.

## 1. Introduction

Timely delivery of status updates is crucial for enabling the operation of many emerging Internet of Things (IoT)-based real-time status updating systems [1]. The concept of AoI was introduced in [2] to quantify the freshness of information available at some node about a physical process as a result of status update receptions over time. In particular, for a single source of information queueing theoretic model in which status updates about a single physical process are generated randomly at a *transmitter node* and are then sent to a *destination node* through a single server, the AoI at the destination was defined in [2] as the following random process: x(t)=t−u(t), where u(t) is the generation time instant of the latest status update received at the destination by a time *t*. Assuming that the AoI process is ergodic, in [2], the stationary average value of the AoI under the first-come-first-serve (FCFS) queueing discipline was derived by leveraging the properties of the AoI’s sample functions and applying appropriate geometric arguments. Although this geometric approach has been considered in a series of subsequent prior works [3,4,5,6,7,8,9,10,11,12,13] to analyze the marginal distributional properties of AoI or peak AoI (an AoI-related metric introduced in [3] to capture the peak values of AoI over time) for adaptations of the queueing model studied in [2], it often requires tedious calculations of joint moments that limit its tractability in analyzing more sophisticated queueing models or disciplines.

Motivated by the above limitations of the geometric approach to AoI analysis, the authors of [14,15] developed an SHS-based framework to allow the analysis of the marginal distributional properties of each AoI process (in a network with multiple AoI processes) through the characterization of its stationary marginal moments and MGF. Furthermore, by using the notion of tensors, the authors of [16] generalized the analysis in [14,15] and developed an SHS-based general framework that facilitates the analysis of the joint distributional properties of an arbitrary set of AoI processes in a network through the characterization of their stationary joint moments and MGFs. In the piecewise linear SHS model with linear reset maps considered in the analyses in [14,15,16], the discrete state of the system q(t) is modeled as a finite-state, continuous-time Markov chain, and the continuous state of the system is modeled using the vector x(t), which contains the AoI or age processes at different nodes in the network. When a transition *l* occurs in q(t) (as a result of status update generation or reception at one of the nodes in the network), the continuous state is updated according to the following linear mapping of x(t): $x^{\prime }\left(t\right)=x\left(t\right)A_{l}$, where $x^{\prime }\left(t\right)$ is the updated version of x(t) and $A_{l}$ is the reset mapping matrix associated with a transition *l*. Additionally, in the absence of a transition in q(t), the age processes in x(t) grow at a unit rate with time, which yields piecewise linear age processes over time. Based on this description of the piecewise linear SHS model with linear reset maps, one can realize that the frameworks in [14,15,16] are not applicable to age analysis in classes of status-updating systems where it is not possible for every transition *l* in q(t) to express the updated value of each age process in the network as a linear combination of the age processes in x(t). A popular class of such systems is the gossip-based status-updating system, where each node in the network randomly shares its information status over time with the other nodes [17,18]. Here, when there is a transition caused by a status update reception at node *j* from node *i*, the updated value of the age process at node *j* is given by the minimum between the values of the age processes at nodes *i* and *j*. As a result, there have been a handful of recent efforts for developing new SHS-based methods that are suitable for age analysis in such gossip networks [19,20]. However, the methods developed thus far have been limited to the characterization of the stationary marginal first moment (average value) of each age process in the network. In this paper, we develop new SHS-based methods that allow the evaluation of the stationary marginal or joint high-order moments of the age processes in gossip networks through the characterization of their stationary marginal or joint MGFs.

### 1.1. Related Work

The literature relevant to this paper can be categorized into the following two categories: (1) prior analyses of AoI applying the SHS approach with linear reset maps and (2) prior analyses of AoI in gossip networks. We now discuss the relevant prior work in these two directions.

*Analyses of AoI applying the SHS approach with linear reset maps*. The SHS approach with linear reset maps developed in [14,15] has been applied to characterize the marginal distributional properties of AoI under a variety of system settings or queueing disciplines [21,22,23,24,25,26,27,28,29,30,31,32,33]. In particular, the average AoI was characterized for single-source systems in [21,22] and multi-source systems in [23,24,25,26,27], whereas the MGF of AoI was derived for single-source systems in [28,29], two-source systems in [30], and multi-source systems in [31,32,33]. Note that a multi-source system refers to the set-up where a transmitter has multiple sources of information generating status updates about multiple physical processes. The authors of [21] derived the average AoI under the last-come-first-serve (LCFS) with preemption in service queueing discipline when the transmitter contained multiple parallel servers. Furthermore, the authors of [22] derived the average AoI under the LCFS with preemption in service queueing discipline when the transmitter contained multiple servers in series or there existed a series of nodes between the transmitter and destination nodes. In [23], the average AoI was characterized under the priority LCFS with preemption in service or waiting queueing model. The authors of [24] derived the average AoI in the presence of packet delivery errors under stationary randomized and round-robin scheduling policies. In [25], the average AoI was characterized under the LCFS with preemption in service queueing discipline when the transmitter contained multiple parallel servers. The authors of [26] analyzed the average AoI for a network in which multiple transmitter-destination pairs contended for the channel using the carrier sense multiple access scheme. In [27] (in [30]), the average AoI (the MGF of AoI) was derived under several source-aware packet management scheduling policies at the transmitter. For the case where the transmitter was powered by energy harvesting (EH), the authors of [28,31] derived the MGF of AoI under several queueing disciplines, including the LCFS with and without preemption in service or waiting strategies. On the other hand, the authors of [16,34] applied their SHS-based framework (developed to allow the analysis of the joint distributional properties of AoI processes in networks) to characterize the joint MGF of an arbitrary set of AoI processes in a multi-source updating system under non-preemptive and source-agnostic or source-aware preemptive-in-service queueing disciplines.

*Analyses of AoI in gossip networks*. There are only a handful of recent works focusing on the analysis or optimization of AoI and its variants in gossip networks [19,20,35,36,37,38,39,40,41]. For a general setting of gossip networks, the author of [19,20] first developed SHS-based methods for the evaluation of the average AoI and the average version age at each node in the network. Note that the version age is a discrete form of AoI defined as the number of versions where the current status of information at a node is out of date compared with the current status of the original source of information. The authors of [35] applied the results of [20] to derive the average version age at each node in several topologies of clustered gossip networks and characterized the average version age scaling as a function of the network size. The authors of [36] extended the SHS-based method developed in [19] for the evaluation of the average AoI in the setting where a timestomping adversary is present and then obtained the average AoI scaling for several network topologies. In [37], each node was assumed to have the ability to estimate the information at the source by applying the majority rule to the information received from the other nodes, and an error metric was introduced to quantify the average percentage of nodes that could accurately obtain the most up-to-date information. The authors of [38,39,40] developed gossip protocols with the objective of improving the average version age scaling. In [41], the problem of optimizing the average version age was formulated as a Markov decision process for a setting where an energy harvesting (EH)-powered sensor was sending status updates to an aggregator with caching capabilities (which served the requests of a gossip network), and the structural properties of the optimal policy were analytically characterized. Different from the analyses in [19,20,35,36,37,38,39,40,41], which were focused on characterizing or optimizing the stationary marginal first moment of AoI or some other AoI-related metrics, this paper is the first to develop SHS-based methods that allow the characterization of the stationary marginal or joint MGFs of AoI processes in gossip networks.

Before delving into more detail about our contributions, it is worth noting that aside from the above queueing theory-based analyses of AoI, there have been efforts to evaluate and optimize AoI or some other AoI-related metrics in a variety of communication systems that deal with time-sensitive information (see [42] for a comprehensive book and [43] for a recent survey). For instance, AoI has been studied in the context of age-optimal transmission scheduling policies [44,45,46,47,48,49,50,51,52], multi-hop networks [53,54,55], broadcast networks [56,57], ultra-reliable low-latency vehicular networks [58], unmanned aerial vehicle (UAV)-assisted communication systems [59,60,61], Internet of Underwater Things networks [62], reconfigurable intelligent surface (RIS)-assisted communication systems [63,64], EH systems [65,66,67,68,69,70,71,72,73,74], large-scale analysis of IoT networks [75,76,77], remote estimation [78,79], information-theoretic analysis [80,81,82,83], timely source coding [84,85], cache updating systems [86,87,88], economic systems [89], and timely communication in federated learning [90,91].

### 1.2. Contributions

A general setting of gossip networks is analyzed in this paper, where a source node forwards its measurements (in the form of status updates) about some observed physical process to a set of monitoring nodes according to independent Poisson processes. Furthermore, each monitoring node sends status updates about its information status (about the process observed by the source) to the other monitoring nodes according to independent Poisson processes. We quantify the freshness of the information available at each monitoring node in terms of AoI. The continuous state of the system is then formed by the AoI or age processes at different monitoring nodes. For this set-up, our main contributions are listed below.

*Developing SHS-based methods for the evaluation of the MGF of age of gossip in networks*. For the general setting of gossip networks described above, we use the SHS framework to characterize (1) the stationary marginal MGF of each age process in the network and (2) the stationary joint MGF of any two arbitrarily selected age processes in the network. In particular, we first construct two classes of test functions (functions whose expected values are quantities of interest) that are suitable for analyzing the marginal or joint MGF. By applying Dynkin’s formula to each test function, we derive two systems of first-order ordinary differential equations characterizing the temporal evolution of the marginal and joint MGFs, from which the stationary marginal and joint MGFs are evaluated. To the best of our knowledge, this paper makes the first attempt at developing SHS-based methods for the characterization of the marginal or joint MGF of age of gossip in networks.

*Analysis of the stationary marginal or joint MGF of age of gossip in three different network topologies*. We apply our developed SHS-based methods to study the marginal or joint distributional properties of age processes in the following three network topologies: (1) a serially-connected topology, (2) a parallelly-connected topology, and (3) a clustered topology. For each of these topologies, we derive close-form expressions for (1) the stationary marginal MGF of the age process at each node and (2) the stationary joint MGFs of all possible pairwise combinations of the age processes.

*System design insights*. Using the MGF expressions derived for each network topology considered in this paper, we obtain closed-form expressions for the following quantities: (1) the stationary marginal first and second moments of each age process, (2) the variance of each age process, and (3) the correlation coefficients between all possible pairwise combinations of the age processes. For these derived quantities, we characterize their structural properties in terms of their convexity and monotonic nature with respect to the status updating rates and further provide asymptotic results showing their behaviors when each of the status updating rates becomes small or large. A key insight drawn from our analysis is that it is crucial to incorporate the higher-order moments of age processes in the implementation or optimization of age-aware gossip networks rather than just relying on the average values of the age processes (as has been performed in the existing literature thus far). This insight promotes the importance of the SHS-based methods developed in this paper for the characterization of the marginal or joint MGFs of different age processes in a general setting of gossip networks.

### 1.3. Organization

The rest of this paper is organized as follows. Section 2 presents the system model and the problem statement. Afterward, in Section 3, we develop the SHS-based methods that allow the evaluation of the stationary marginal or joint high-order moments of the age processes in gossip networks through the characterization of their stationary marginal or joint MGFs. Section 4 applies the SHS-based methods developed in Section 3 to derive the marginal or joint MGFs of age processes at different nodes in three different connected network settings. For each considered connected network setting, we further use the derived MGF expressions to obtain the marginal or joint high-order statistics of age processes such as the variance of each age process and the correlation coefficients between all possible pairwise combinations of the age processes. Finally, Section 5 concludes the paper.

## 2. System Model and Problem Statement

We consider a general setting of gossip networks where a source node (referred to as node 0) provides its measurements about some observed physical process for a set of nodes N={1,2,⋯,N} in the form of status updates. In particular, all the nodes in N are tracking the age of the process observed by the source, and the status updates sent by node 0 to node j∈N are assumed to follow an independent Poisson process with a rate $\lambda_{0j}$. Aside from that, node i∈N sends updates about its information status (about the process observed by the source) to each node j∈N\{i} according to an independent Poisson process with a rate $\lambda_{ij}$. When $\lambda_{ij}>0$, we say that nodes *i* and *j* are connected to each other. Since we allow each $\lambda_{ij}$ (i∈{0}∪N and j∈N) to take a value in [0,∞], we refer to the above setting as an arbitrarily connected gossip network. Note that this gossip network setting is of interest in many practical networks, such as low-latency vehicular networks and UAV-assisted communication networks. The freshness of status of the information available at each node is quantified in terms of AoI. Let $x_{i}\left(t\right)$ denote the AoI process (or equivalently the age process) at node i∈N. Assuming that node 0 always maintains a fresh status of information about the observed physical process, the age or AoI at node j∈N is reset to zero whenever it receives a status update from node 0. Furthermore, when node j∈N receives a status update from node i∈N\{j} at time *t*, its age $x_{j}\left(t\right)$ is reset to the age of node *i*$x_{i}\left(t\right)$ only if $x_{i}\left(t\right)$ is smaller than $x_{j}\left(t\right)$. To summarize, when node j∈N receives a status update from node i∈{0}∪N, the age at node k∈N is updated as follows:

(1) $$\begin{array}{c} x_{k}^{\prime }\left(t\right)=\left\{\begin{array}{cc} 0, & \mathrm{if}\;\;i=0\;\;\mathrm{and}\;\;k=j,\\ \min\left[x_{j}\left(t\right),x_{i}\left(t\right)\right], & \mathrm{if}\;\;i\in N\;\;\mathrm{and}\;\;k=j,\\ x_{k}\left(t\right), & \mathrm{otherwise}. \end{array}\right. \end{array}$$

For an arbitrary set S⊆N, define $x_{S}\left(t\right)=\min_{i\in S}x_{i}\left(t\right)$ as the age or AoI process associated with *S* (or simply the age or AoI of *S*). For the above gossip network setting, the method developed in [19] has been limited to the characterization of the stationary marginal first moment of $x_{S}\left(t\right)$ (i.e., the stationary average value of $x_{S}\left(t\right)$). In this paper, our prime objective is to develop a method that allows characterizing (1) the stationary marginal higher-order moments of $x_{S}\left(t\right)$ and (2) the stationary joint high-order moments of the two age processes associated with two arbitrary sets $S_{1}$ and $S_{2}$ (i.e., $x_{S_{1}}\left(t\right)$ and $x_{S_{2}}\left(t\right)$, respectively). Note that we do not place any restrictions on the construction of $S_{1}$ or $S_{2}$. For instance, they could even have common elements. Formally, we aim at characterizing the stationary marginal MGF of $x_{S}\left(t\right)$ and the stationary joint MGF of $x_{S_{1}}\left(t\right)$ and $x_{S_{2}}\left(t\right)$, which are of the following forms: $\lim_{t\to \infty }E\left[\exp\left[nx_{S}\left(t\right)\right]\right]$ and $\lim_{t\to \infty }E\left[\exp\left[n_{1}x_{S_{1}}\left(t\right)+n_{2}x_{S_{2}}\left(t\right)\right]\right]$, respectively, where $n,n_{1},n_{2}\in R$ and $S,S_{1},S_{2}\subseteq N$. As will be evident from the technical sections shortly, the characterization of such MGFs allows one to derive the marginal or joint high-order statistics of the AoI processes at different nodes in the network, such as the variance of each AoI process and the correlation coefficients between all possible pairwise combinations of the AoI processes. Given the generality of the system setting considered in this paper, the importance of our method lies in the fact that it is applicable to the marginal or joint analysis of AoI processes for an arbitrary structured gossip network setting.

## 3. MGF Analysis of Age in Arbitrarily Connected Gossip Networks

In this section, we first formulate the problem at hand as an SHS. We then use the SHS framework to characterize (1) the stationary marginal MGF of the age process associated with an arbitrary set S⊆N (i.e., $x_{S}\left(t\right)$) and (2) the stationary joint MGF of the two age processes associated with two arbitrary sets $S_{1}\subseteq N$ and $S_{2}\subseteq N$ (i.e., $x_{S_{1}}\left(t\right)$ and $x_{S_{2}}\left(t\right)$, respectively) for the arbitrarily connected gossip network setting described in Section 2.

The SHS framework is used to analyze hybrid queueing systems that can be modeled by a combination of discrete and continuous state parameters. For the gossip network setting considered in this paper, the continuous state of the system is modeled using the row vector $x\left(t\right)=\left[x_{1}\left(t\right)\;x_{2}\left(t\right)\;\cdots \;x_{N}\left(t\right)\right]$ containing the AoI or age processes at different nodes in the network. Furthermore, since the status updates sent by each node in the network to the other nodes are assumed to follow independent Poisson processes, it is sufficient to model the discrete state of the system as a singleton set. To complete the description of an SHS, one needs to define a set of transitions L along with the continuous and discrete states of the system. This set L refers to changes in either the continuous state or the discrete state. Since the discrete state of the SHS under consideration is a singleton set, the set L corresponds to only the changes in the continuous state of the system. In our system setting, a change in the continuous state of the system occurs when there is a status update reception at some node in the network. Furthermore, as long as there is no status update reception at any of the nodes, the AoI or age at each node grows linearly with time (which yields piecewise linear age processes over time); in other words, $\dot{x}\left(t\right)=1_{N}$, where $1_{N}$ is the row vector $\left[1\;\cdots \;1\right]\in R^{1\times N}$. By inspecting the age updating rule in (1), the set L can be defined as follows:

(2) $$\begin{array}{c} L=\{(0,j):j\in N\}\cup \{(i,j):i,j\in N\}. \end{array}$$

For the above SHS-based formulation, we derive two systems of linear equations for evaluating the stationary marginal MGF $\lim_{t\to \infty }E\left[\exp\left[nx_{S}\left(t\right)\right]\right]$ and the stationary joint MGF $\lim_{t\to \infty }E\left[\exp\left[n_{1}x_{S_{1}}\left(t\right)+n_{2}x_{S_{2}}\left(t\right)\right]\right]$. The description of these systems of equations and the presentation of the subsequent results require defining the following quantities:

(3) $$\begin{array}{c} v_{S}^{\left(n\right)}\left(t\right)=E\left[\exp\left[nx_{S}\left(t\right)\right]\right],\;{\bar{v}}_{S}^{\left(n\right)}=\lim_{t\to \infty }v_{S}^{\left(n\right)},\;\;\forall S\subseteq N, \end{array}$$

(4) $$\begin{array}{c} v_{S_{1},S_{2}}^{\left(n_{1},n_{2}\right)}\left(t\right)=E\left[\exp\left[n_{1}x_{S_{1}}\left(t\right)+n_{2}x_{S_{2}}\left(t\right)\right]\right],\;{\bar{v}}_{S_{1},S_{2}}^{\left(n_{1},n_{2}\right)}=\lim_{t\to \infty }v_{S_{1},S_{2}}^{\left(n_{1},n_{2}\right)},\;\;\forall S_{1},S_{2}\subseteq N, \end{array}$$

(5) $$\begin{array}{c} v_{S}^{\left(m\right)}\left(t\right)=E\left[x_{S}^{m}\left(t\right)\right],\;{\bar{v}}_{S}^{\left(m\right)}=\lim_{t\to \infty }v_{S}^{\left(m\right)},\;\;\forall S\subseteq N, \end{array}$$

(6) $$\begin{array}{c} v_{S_{1},S_{2}}^{\left(m_{1},m_{2}\right)}\left(t\right)=E\left[x_{S_{1}}^{m_{1}}\left(t\right)x_{S_{2}}^{m_{2}}\left(t\right)]\right],\;{\bar{v}}_{S_{1},S_{2}}^{\left(m_{1},m_{2}\right)}=\lim_{t\to \infty }v_{S_{1},S_{2}}^{\left(m_{1},m_{2}\right)},\;\;\forall S_{1},S_{2}\subseteq N, \end{array}$$

where $v_{S}^{\left(m\right)}$ is the marginal *m*th moment of the age process $x_{S}\left(t\right)$ and $v_{S_{1},S_{2}}^{\left(m_{1},m_{2}\right)}$ is the joint moment of the two age processes $x_{S_{1}}\left(t\right)$ and $x_{S_{2}}\left(t\right)$. From (3) and (5), $v_{S}^{\left(1\right)}\left(t\right)$ may generally refer to $v_{S}^{\left(n\right)}{\left(t\right)|}_{n=1}$ or $v_{S}^{\left(m\right)}{\left(t\right)|}_{m=1}$. To eliminate this conflict, the convention that $v_{S}^{\left(i\right)}\left(t\right)$ for an integer *i* refers to $v_{S}^{\left(m\right)}\left(t\right)$ at m=i is maintained here. The previous argument also applies to $v_{S_{1},S_{2}}^{\left(n_{1},n_{2}\right)}\left(t\right)$ and $v_{S_{1},S_{2}}^{\left(m_{1},m_{2}\right)}\left(t\right)$ in (4) and (6), respectively, where $v_{S_{1},S_{2}}^{\left(i,j\right)}\left(t\right)$, for integers *i* and *j*, refers to $v_{S_{1},S_{2}}^{\left(m_{1},m_{2}\right)}\left(t\right)$ at $m_{1}=i$ and $m_{2}=j$. Furthermore, following the notations in [19], we define the update rate of node *i* into set *S* and the set of updating neighbors of *S* as

(7) $$\begin{array}{c} \lambda_{i}\left(S\right)=\left\{\begin{array}{cc} \sum_{j\in S}\lambda_{i,j},\; & \mathrm{if}\;i\notin S,\\ 0,\; & \mathrm{otherwise}, \end{array}\right. \end{array}$$

(8) $$\begin{array}{c} N\left(S\right)=\{i\in N:\lambda_{i}\left(S\right)>0\}. \end{array}$$

We are now ready to present the two systems of linear equations for the evaluation of ${\bar{v}}_{S}^{\left(n\right)}$ and ${\bar{v}}_{S_{1},S_{2}}^{\left(n_{1},n_{2}\right)}$ in the following two theorems:

**Theorem 1.**  *For an arbitrarily connected gossip network, there exists a threshold δ>0 such that for n∈[0,δ), the stationary marginal MGF of AoI of set S⊆N is given by*

(9) $$\begin{array}{c} {\bar{v}}_{S}^{\left(n\right)}=\frac{\lambda_{0}\left(S\right)+\sum_{i\in N(S)}\lambda_{i}\left(S\right){\bar{v}}_{S\cup \{i\}}^{\left(n\right)}}{\lambda_{0}\left(S\right)+\sum_{i\in N(S)}\lambda_{i}\left(S\right)-n}. \end{array}$$

*Furthermore, for m≥1, the stationary marginal m-th moment of AoI of set S⊆N is given by*

(10) $$\begin{array}{c} {\bar{v}}_{S}^{\left(m\right)}=\frac{m{\bar{v}}_{S}^{\left(m-1\right)}+\sum_{i\in N(S)}\lambda_{i}\left(S\right){\bar{v}}_{S\cup \{i\}}^{\left(m\right)}}{\lambda_{0}\left(S\right)+\sum_{i\in N(S)}\lambda_{i}\left(S\right)}. \end{array}$$

**Proof of Theorem 1.**  See Appendix A. □

**Theorem 2.**  *For an arbitrarily connected gossip network, there exists a threshold δ>0 such that for $0\leq n_{1}+n_{2}<\delta$, the stationary joint MGF of the two AoI processes associated with the two sets $S_{1}$ and $S_{2}$ is given by*

(11) $$\begin{array}{cc} {\bar{v}}_{S_{1},S_{2}}^{\left(n_{1},n_{2}\right)}= & \frac{1}{\lambda_{0}\left(S_{1}\cup S_{2}\right)+\sum_{i\in N\backslash (S_{1}\cap S_{2})}\lambda_{i}\left(S_{1}\cap S_{2}\right)+\sum_{i\in N\backslash S_{1}}\lambda_{i}\left(S_{1}\backslash S_{2}\right)+\sum_{i\in N\backslash S_{2}}\lambda_{i}\left(S_{2}\backslash S_{1}\right)-\left(n_{1}+n_{2}\right)}\times [\lambda_{0}\left(S_{1}\cap S_{2}\right)\\ & +\lambda_{0}\left(S_{1}\backslash S_{2}\right){\bar{v}}_{S_{2}}^{\left(n_{2}\right)}+\lambda_{0}\left(S_{2}\backslash S_{1}\right){\bar{v}}_{S_{1}}^{\left(n_{1}\right)}+\sum_{i\in N\backslash S_{1}}\lambda_{i}\left(S_{1}\backslash S_{2}\right){\bar{v}}_{S_{1}\cup \left\{i\right\},S_{2}}^{\left(n_{1},n_{2}\right)}+\sum_{i\in N\backslash S_{2}}\lambda_{i}\left(S_{2}\backslash S_{1}\right){\bar{v}}_{S_{1},S_{2}\cup \left\{i\right\}}^{\left(n_{1},n_{2}\right)}\\ \; & +\sum_{i\in N\backslash (S_{1}\cup S_{2})}\lambda_{i}\left(S_{1}\cap S_{2}\right){\bar{v}}_{S_{1}\cup \left\{i\right\},S_{2}\cup \left\{i\right\}}^{\left(n_{1},n_{2}\right)}+\sum_{i\in S_{1}\backslash S_{2}}\lambda_{i}\left(S_{1}\cap S_{2}\right){\bar{v}}_{S_{1},S_{2}\cup \left\{i\right\}}^{\left(n_{1},n_{2}\right)}+\sum_{i\in S_{2}\backslash S_{1}}\lambda_{i}\left(S_{1}\cap S_{2}\right){\bar{v}}_{S_{1}\cup \left\{i\right\},S_{2}}^{\left(n_{1},n_{2}\right)}]. \end{array}$$

*Furthermore, for $m_{1},m_{2}\geq 1$, the stationary joint $\left(m_{1},m_{2}\right)$-th moment of the AoI processes associated with the two sets $S_{1}$ and $S_{2}$ is given by*

(12) $$\begin{array}{cc} {\bar{v}}_{S_{1},S_{2}}^{\left(m_{1},m_{2}\right)}= & \frac{1}{\lambda_{0}\left(S_{1}\cup S_{2}\right)+\sum_{i\in N\backslash (S_{1}\cap S_{2})}\lambda_{i}\left(S_{1}\cap S_{2}\right)+\sum_{i\in N\backslash S_{1}}\lambda_{i}\left(S_{1}\backslash S_{2}\right)+\sum_{i\in N\backslash S_{2}}\lambda_{i}\left(S_{2}\backslash S_{1}\right)}\times [m_{1}{\bar{v}}_{S_{1},S_{2}}^{\left(m_{1}-1,m_{2}\right)}\\ & +m_{2}{\bar{v}}_{S_{1},S_{2}}^{\left(m_{1},m_{2}-1\right)}+\sum_{i\in N\backslash S_{1}}\lambda_{i}\left(S_{1}\backslash S_{2}\right){\bar{v}}_{S_{1}\cup \left\{i\right\},S_{2}}^{\left(m_{1},m_{2}\right)}+\sum_{i\in N\backslash S_{2}}\lambda_{i}\left(S_{2}\backslash S_{1}\right){\bar{v}}_{S_{1},S_{2}\cup \left\{i\right\}}^{\left(m_{1},m_{2}\right)}+\sum_{i\in N\backslash (S_{1}\cup S_{2})}\lambda_{i}\left(S_{1}\cap S_{2}\right){\bar{v}}_{S_{1}\cup \left\{i\right\},S_{2}\cup \left\{i\right\}}^{\left(m_{1},m_{2}\right)}\\ \; & +\sum_{i\in S_{1}\backslash S_{2}}\lambda_{i}\left(S_{1}\cap S_{2}\right){\bar{v}}_{S_{1},S_{2}\cup \left\{i\right\}}^{\left(m_{1},m_{2}\right)}+\sum_{i\in S_{2}\backslash S_{1}}\lambda_{i}\left(S_{1}\cap S_{2}\right){\bar{v}}_{S_{1}\cup \left\{i\right\},S_{2}}^{\left(m_{1},m_{2}\right)}]. \end{array}$$

**Proof of Theorem 2.**  See Appendix B. □

**Remark 1.**  *Note that the stationary marginal MGF of $S_{1}$ or $S_{2}$ can be obtained from the stationary joint MGF in (11). In particular, when $n_{2}=0$ and $S_{2}=⌀$, ${\bar{v}}_{S_{1},S_{2}}^{\left(n_{1},n_{2}\right)}$ reduces to*

(13) $$\begin{array}{c} {\bar{v}}_{S_{1},⌀}^{\left(n_{1},0\right)}=\frac{\lambda_{0}\left(S_{1}\right)+\sum_{i\in N(S_{1})}\lambda_{i}\left(S_{1}\right){\bar{v}}_{S_{1}\cup \left\{i\right\}}^{\left(n\right)}}{\lambda_{0}\left(S_{1}\right)+\sum_{i\in N(S_{1})}\lambda_{i}\left(S_{1}\right)-n_{1}}\overset{\left(a\right)}{=}{\bar{v}}_{S_{1}}^{\left(n_{1}\right)}, \end{array}$$

*where step (a) follows from (9). Similarly, one can observe that ${\bar{v}}_{⌀,S_{2}}^{\left(0,n_{2}\right)}={\bar{v}}_{S_{2}}^{\left(n_{2}\right)}$.*

*Furthermore, when m=1, (10) reduces to ([19] Theorem 1) characterizing the stationary marginal first moment of the AoI of set S⊆N.*

It is worth highlighting that the generality of Theorems 1 and 2 lies in the fact that they allow one to investigate the stationary marginal or joint MGFs of the age processes at different nodes in an arbitrarily connected gossip network. This opens the door for the application of Theorems 1 and 2 to characterize the marginal or joint high-order moments of age processes for different configurations or topologies of gossip networks studied in the literature, which have only been analyzed in terms of the marginal first moments of age processes (i.e., average age values) until now. Furthermore, the expressions in (10) and (12) provide a straightforward way for the numerical evaluation of the stationary marginal or joint high-order moments.

## 4. Applications of Theorems 1 and 2

**Figure 1:**
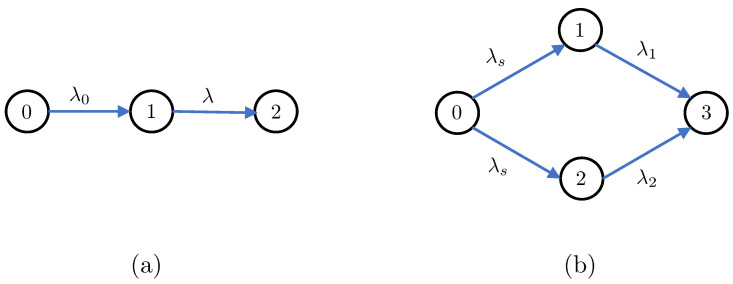
(**a**) A serially-connected network setting. (**b**) A parallelly-connected network setting.

**Figure 2:**
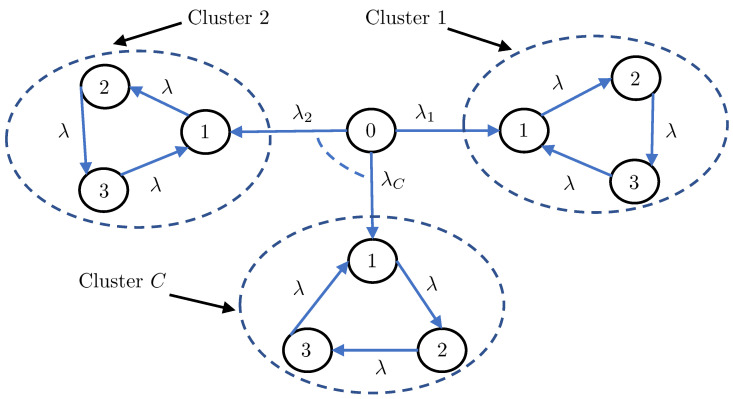
A clustered gossip network topology consisting of *C* clusters such that the status updating rate from node 0 to the *c*-th cluster is $\lambda_{c}$.

In this section, we first apply Theorems 1 and 2 to understand the distributional properties of the age processes in the two canonical settings depicted in Figure 1 (i.e., the the serially and parallelly-connected network settings). We then aim to analyze a more complicated network setting, which was chosen to be the clustered gossip network topology depicted in Figure 2. Our choice for the clustered gossip network setting was inspired by the recent interest in analyzing its different topologies in terms of the marginal first moment of each age process (average age) in the network [35].

### 4.1. Serially-Connected Networks

**Theorem 3.**  *For the serially-connected network in Figure 1a, the stationary marginal MGFs of the AoI processes at nodes 1 and 2 are respectively given by*

(14) $$\begin{array}{c} {\bar{v}}_{\left\{1\right\}}^{\left(n\right)}=\frac{\lambda_{0}}{\lambda_{0}-n}, \end{array}$$

(15) $$\begin{array}{c} {\bar{v}}_{\left\{2\right\}}^{\left(n\right)}=\frac{\lambda_{0}\lambda }{\left(\lambda_{0}-n\right)\left(\lambda -n\right)}. \end{array}$$

*Additionally, the stationary joint MGF of the two AoI processes at nodes 1 and 2 is given by*

(16) $$\begin{array}{c} {\bar{v}}_{\left\{2\},\{1\right\}}^{\left(n_{1},n_{2}\right)}=\frac{\lambda_{0}\lambda }{\lambda_{0}+\lambda -\left(n_{1}+n_{2}\right)}\left(\frac{\lambda_{0}}{\left(\lambda_{0}-n_{1}\right)\left(\lambda -n_{1}\right)}+\frac{1}{\lambda_{0}-\left(n_{1}+n_{2}\right)}\right). \end{array}$$

**Proof of Theorem 3.**  See Appendix C. □

**Proposition 1.**  *For the serially-connected network in Figure 1a, the first moment, second moment, and variance of the AoI process at each node are given by*

(17) $$\begin{array}{c} {\bar{v}}_{\left\{1\right\}}^{\left(1\right)}=\lambda_{0}^{-1},\;\;{\bar{v}}_{\left\{1\right\}}^{\left(2\right)}=2\lambda_{0}^{-2},\;\;\mathrm{var}\left[x_{1}\left(t\right)\right]=\lambda_{0}^{-2}, \end{array}$$

(18) $$\begin{array}{c} {\bar{v}}_{\left\{2\right\}}^{\left(1\right)}=\frac{1}{\lambda_{0}}+\frac{1}{\lambda },\;\;{\bar{v}}_{\left\{2\right\}}^{\left(2\right)}=2\left(\frac{1}{\lambda_{0}^{2}}+\frac{1}{\lambda_{0}\lambda }+\frac{1}{\lambda^{2}}\right),\;\;\mathrm{var}\left[x_{2}\left(t\right)\right]=\frac{1}{\lambda_{0}^{2}}+\frac{1}{\lambda^{2}}. \end{array}$$

*Furthermore, the correlation coefficient between the AoI processes at nodes 1 and 2 can be expressed as*

(19) $$\begin{array}{c} \mathrm{cor}\left[x_{1}\left(t\right),x_{2}\left(t\right)\right]=\frac{\lambda^{2}}{\left(\lambda_{0}+\lambda \right)\sqrt{\lambda_{0}^{2}+\lambda^{2}}}. \end{array}$$

**Proof of Proposition 1.**  See Appendix D. □

**Remark 2.**  *Note that the expressions of the stationary marginal MGFs in Theorem 3 and the stationary marginal moments in Proposition 1 match their corresponding ones for the preemptive line networks analyzed in [15].*

**Remark 3.**  *Note that the stationary moments and variance of the age process at node 1 in (17) are univariate functions of $\lambda_{0}$. This happens because node 1 is directly connected to node 0. This argument will also apply to: (i) the expressions derived for the age processes at nodes 1 and 2 in the parallelly-connected network in Figure 1b, and (ii) the expressions derived for the age process at node 1 inside each cluster of the clustered gossip network in Figure 2.*

**Figure 3:**
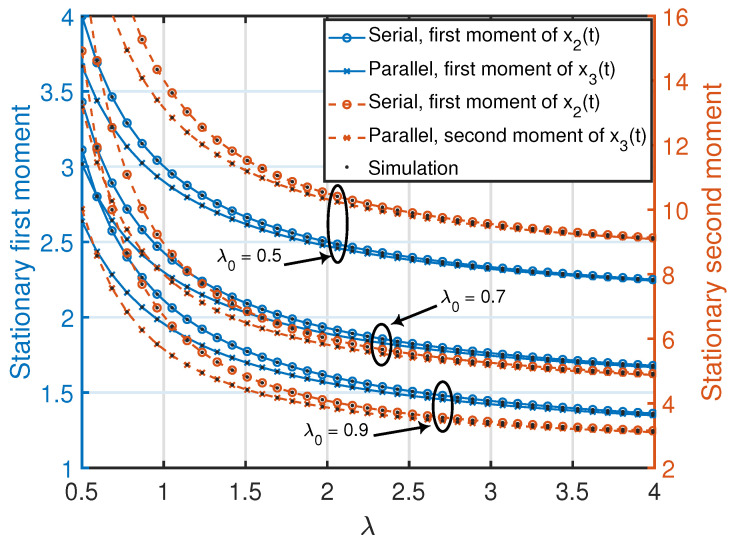
Stationary first and second moments of age processes in the serially and parallelly-connected network settings. We set $\lambda_{s}=0.5\lambda_{0}$ and $\lambda =\lambda_{1}=\lambda_{2}$. The simulated curves are obtained from the numerical evaluation of the stationary marginal moments using (10) in Theorem 1.

**Remark 4.**  *Note that the stationary moments and variance of the age process at node 2 in (18) are invariant to exchanging λ and $\lambda_{0}$. These quantities are also jointly convex functions in $\left(\lambda_{0},\lambda \right)$, where the minimum value (zero) of each function is achieved at $\lambda_{0}=\lambda =\infty$. Furthermore, for a given λ or $\lambda_{0}$, each quantity in (18) is a monotonically non-increasing function with respect to $\lambda_{0}$ or λ. This can also be observed in Figure 3.*

**Figure 4:**
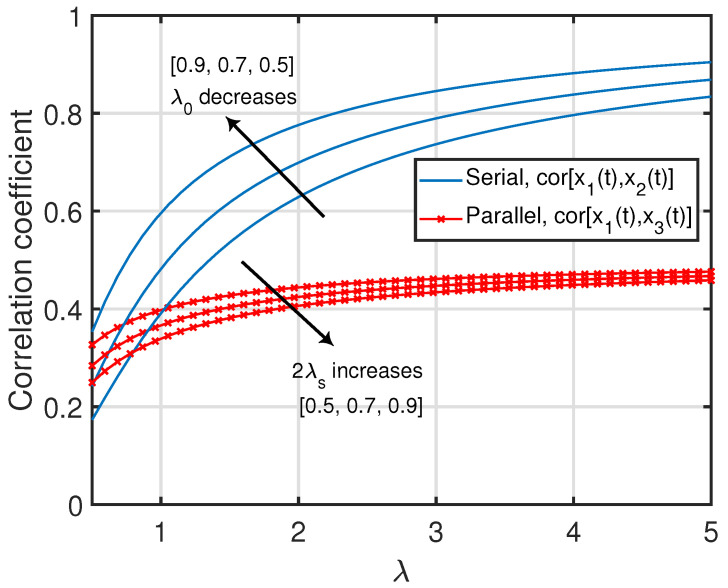
Correlation coefficients between age processes in the serially and parallelly-connected network settings.

**Remark 5.**  *For a given λ, $\mathrm{cor}[x_{1}\left(t\right),x_{2}\left(t\right)]$ in (19) monotonically decreases as a function of $\lambda_{0}$ in the form $\lim_{\lambda_{0}\to 0}\mathrm{cor}\left[x_{1}\left(t\right),x_{2}\left(t\right)\right]=1$ until it approaches $\lim_{\lambda_{0}\to \infty }\mathrm{cor}\left[x_{1}\left(t\right),x_{2}\left(t\right)\right]=0$. On the other hand, for a given $\lambda_{0}$, $\mathrm{cor}[x_{1}\left(t\right),x_{2}\left(t\right)]$ monotonically increases as a function of λ in the form $\lim_{\lambda \to 0}\mathrm{cor}\left[x_{1}\left(t\right),x_{2}\left(t\right)\right]=0$ until it approaches $\lim_{\lambda \to \infty }\mathrm{cor}\left[x_{1}\left(t\right),x_{2}\left(t\right)\right]=1$. This can also be observed in Figure 4.*

### 4.2. Parallelly-Connected Networks

**Theorem 4.**  *For the parallelly-connected network in Figure 1b, the stationary marginal MGFs of the AoI processes at nodes 1, 2, and 3 are given by*

(20) $$\begin{array}{c} {\bar{v}}_{\left\{1\right\}}^{\left(n\right)}={\bar{v}}_{\left\{2\right\}}^{\left(n\right)}=\frac{\lambda_{s}}{\lambda_{s}-n}, \end{array}$$

(21) $$\begin{array}{c} \;{\bar{v}}_{\left\{3\right\}}^{\left(n\right)}=\frac{\lambda_{s}\left(2\lambda_{s}-n\right)\left[\lambda_{1}\left(\lambda_{s}+\lambda_{1}-n\right)+\lambda_{2}\left(\lambda_{s}+\lambda_{2}-n\right)\right]+2\lambda_{s}\lambda_{1}\lambda_{2}\left(2\lambda_{s}+\lambda_{1}+\lambda_{2}-2n\right)}{\left(2\lambda_{s}-n\right)\left(\lambda_{1}+\lambda_{2}-n\right)\left(\lambda_{s}+\lambda_{1}-n\right)\left(\lambda_{s}+\lambda_{2}-n\right)}. \end{array}$$

*Additionally, the stationary joint MGF of the two AoI processes at nodes 1 and 3 is given by*

(22) $$\begin{array}{cc} {\bar{v}}_{\left\{3\},\{1\right\}}^{\left(n_{1},n_{2}\right)}= & \frac{\sum_{i=1}^{4}\alpha_{i}\left(n_{1},n_{2}\right)}{\left[\lambda_{s}+\lambda_{1}+\lambda_{2}-\left(n_{1}+n_{2}\right)\right]\left[2\lambda_{s}+\lambda_{1}-\left(n_{1}+n_{2}\right)\right]\left[2\lambda_{s}-\left(n_{1}+n_{2}\right)\right]\left[\lambda_{s}+\lambda_{2}-\left(n_{1}+n_{2}\right)\right]}\\ & \times \frac{1}{\left(\lambda_{s}-n_{2}\right)\left(\lambda_{1}+\lambda_{2}-n_{1}\right)\left(2\lambda_{s}-n_{1}\right)\left(\lambda_{s}+\lambda_{2}-n_{1}\right)\left(\lambda_{s}+\lambda_{1}-n_{1}\right)}, \end{array}$$

*where*

(23) $$\begin{array}{cc} \alpha_{1}\left(n_{1},n_{2}\right)= & \lambda_{s}^{2}\left(\lambda_{s}-n_{2}\right)\left[\lambda_{s}+\lambda_{2}-\left(n_{1}+n_{2}\right)\right]\left[2\lambda_{s}+\lambda_{1}-\left(n_{1}+n_{2}\right)\right]\left[2\lambda_{s}-\left(n_{1}+n_{2}\right)\right]\\ \; & \times \left[\left(2\lambda_{2}-n_{1}\right)\left[\lambda_{1}\left(\lambda_{s}+\lambda_{1}-n_{1}\right)+\lambda_{2}\left(\lambda_{s}+\lambda_{2}-n_{1}\right)\right]+2\lambda_{1}\lambda_{2}\left(2\lambda_{s}+\lambda_{1}+\lambda_{2}-2n_{1}\right)\right], \end{array}$$

(24) $$\begin{array}{c} \alpha_{2}\left(n_{1},n_{2}\right)=\lambda_{s}^{2}\lambda_{2}\left(\lambda_{1}+\lambda_{2}-n_{1}\right)\left(2\lambda_{s}-n_{1}\right)\left(\lambda_{s}+\lambda_{1}-n_{1}\right)\left(\lambda_{s}+\lambda_{2}-n_{1}\right)\left[2\lambda_{s}+\lambda_{1}-\left(n_{1}+n_{2}\right)\right]\left[\lambda_{s}+\lambda_{1}+\lambda_{2}-\left(n_{1}+n_{2}\right)\right], \end{array}$$

(25) $$\begin{array}{c} \alpha_{3}\left(n_{1},n_{2}\right)=\lambda_{s}^{2}\lambda_{2}\left(\lambda_{1}+\lambda_{2}-n_{1}\right)\left(\lambda_{s}+\lambda_{2}-n_{1}\right)\left(2\lambda_{s}+2\lambda_{1}-n_{1}\right)\left(\lambda_{s}-n_{2}\right)\left[\lambda_{s}+\lambda_{2}-\left(n_{1}+n_{2}\right)\right]\left[2\lambda_{s}-\left(n_{1}+n_{2}\right)\right], \end{array}$$

(26) $$\begin{array}{cc} \alpha_{4}\left(n_{1},n_{2}\right)= & \lambda_{s}\lambda_{1}\left(\lambda_{s}-n_{2}\right)\left(\lambda_{1}+\lambda_{2}-n_{1}\right)\left(2\lambda_{s}-n_{1}\right)\left(\lambda_{s}+\lambda_{2}-n_{1}\right)\left(\lambda_{s}+\lambda_{1}-n_{1}\right)\\ \; & \times \left[\left[2\lambda_{s}+\lambda_{1}-\left(n_{1}+n_{2}\right)\right]\left[2\lambda_{s}+\lambda_{2}-\left(n_{1}+n_{2}\right)\right]+\lambda_{2}\left[\lambda_{s}+\lambda_{2}-\left(n_{1}+n_{2}\right)\right]\right]. \end{array}$$

**Proof of Theorem 4.**  See Appendix E. □

**Proposition 2.**  *For the parallelly-connected network in Figure 1b, the first moment, second moment, and variance of the AoI process at each node are given by*

(27) $$\begin{array}{c} {\bar{v}}_{\left\{1\right\}}^{\left(1\right)}={\bar{v}}_{\left\{2\right\}}^{\left(1\right)}=\lambda_{s}^{-1},\;\;{\bar{v}}_{\left\{1\right\}}^{\left(2\right)}={\bar{v}}_{\left\{2\right\}}^{\left(2\right)}=2\lambda_{s}^{-2},\;\;\mathrm{var}\left[x_{1}\left(t\right)\right]=\mathrm{var}\left[x_{2}\left(t\right)\right]=\lambda_{s}^{-2}, \end{array}$$

(28) $$\begin{array}{c} {\bar{v}}_{\left\{3\right\}}^{\left(1\right)}=\frac{2\lambda_{s}\left(\lambda_{s}+\lambda_{1}\right)\left(\lambda_{s}+\lambda_{2}\right)+\lambda_{1}\left(2\lambda_{s}+\lambda_{2}\right)\left(\lambda_{s}+\lambda_{1}\right)+\lambda_{2}\left(2\lambda_{s}+\lambda_{1}\right)\left(\lambda_{s}+\lambda_{2}\right)}{2\lambda_{s}\left(\lambda_{s}+\lambda_{1}\right)\left(\lambda_{s}+\lambda_{2}\right)\left(\lambda_{1}+\lambda_{2}\right)}, \end{array}$$

(29) $$\begin{array}{c} {\bar{v}}_{\left\{3\right\}}^{\left(2\right)}=\frac{\sum_{i=0}^{6}\gamma_{i}\lambda_{s}^{i}}{2\lambda_{s}^{2}{\left(\lambda_{1}+\lambda_{2}\right)}^{2}{\left(\lambda_{s}+\lambda_{1}\right)}^{2}{\left(\lambda_{s}+\lambda_{2}\right)}^{2}}, \end{array}$$

(30) $$\begin{array}{c} \mathrm{var}\left[x_{3}\left(t\right)\right]=\frac{\sum_{i=0}^{6}\eta_{i}\lambda_{s}^{i}}{4\lambda_{s}^{2}{\left(\lambda_{1}+\lambda_{2}\right)}^{2}{\left(\lambda_{s}+\lambda_{1}\right)}^{2}{\left(\lambda_{s}+\lambda_{2}\right)}^{2}}, \end{array}$$

*where*

$$\begin{array}{c} \gamma_{6}=4,\;\gamma_{5}=12\left(\lambda_{1}+\lambda_{2}\right),\;\gamma_{4}=4\left[4{\left(\lambda_{1}+\lambda_{2}\right)}^{2}+\lambda_{1}\lambda_{2}\right],\;\gamma_{3}=12{\left(\lambda_{1}+\lambda_{2}\right)}^{3},\;\\ \gamma_{2}={\left(\lambda_{1}+\lambda_{2}\right)}^{2}\left[4{\left(\lambda_{1}+\lambda_{2}\right)}^{2}+\lambda_{1}\lambda_{2}\right],\;\gamma_{1}=3\lambda_{1}\lambda_{2}{\left(\lambda_{1}+\lambda_{2}\right)}^{3},\gamma_{0}=\lambda_{1}^{2}\lambda_{2}^{2}{\left(\lambda_{1}+\lambda_{2}\right)}^{2},\\ \eta_{6}=4,\;\eta_{5}=8\left(\lambda_{1}+\lambda_{2}\right),\;\eta_{4}=8\left[{\left(\lambda_{1}+\lambda_{2}\right)}^{2}+\lambda_{1}\lambda_{2}\right],\;\eta_{3}=4\left(\lambda_{1}+\lambda_{2}\right)\left(2\lambda_{1}^{2}+3\lambda_{1}\lambda_{2}+2\lambda_{2}^{2}\right),\;\\ \eta_{2}=2{\left(\lambda_{1}+\lambda_{2}\right)}^{2}\left(2\lambda_{1}^{2}+\lambda_{1}\lambda_{2}+2\lambda_{2}^{2}\right),\;\eta_{1}=2\lambda_{1}\lambda_{2}{\left(\lambda_{1}+\lambda_{2}\right)}^{3},\eta_{0}=\lambda_{1}^{2}\lambda_{2}^{2}{\left(\lambda_{1}+\lambda_{2}\right)}^{2}. \end{array}$$

*Furthermore, the correlation coefficient between the AoI processes at nodes 1 and 3 can be expressed as*

(31) $$\begin{array}{cc} \mathrm{cor}\left[x_{1}\left(t\right),x_{3}\left(t\right)\right]= & \frac{\lambda_{1}\left(\lambda_{1}+\lambda_{2}\right)}{2\left(\lambda_{s}+\lambda_{1}+\lambda_{2}\right)\left(2\lambda_{s}+\lambda_{1}\right)\left(\lambda_{s}+\lambda_{2}\right)\sqrt{\sum_{i=0}^{6}\delta_{i}\lambda_{s}^{i}}}\\ \; & \times \left[8\lambda_{s}^{4}+\lambda_{s}^{3}\left(12\lambda_{1}+7\lambda_{2}\right)+2\lambda_{s}^{2}\left(\lambda_{1}+2\lambda_{2}\right)\left(2\lambda_{1}+\lambda_{2}\right)+\lambda_{s}\lambda_{2}\left(3\lambda_{1}^{2}+5\lambda_{1}\lambda_{2}+\lambda_{2}^{2}\right)+\lambda_{1}\lambda_{2}^{2}\left(\lambda_{1}+\lambda_{2}\right)\right], \end{array}$$

*where*

$$\begin{array}{c} \delta_{6}=4,\;\delta_{5}=8\left(\lambda_{1}+\lambda_{2}\right),\;\delta_{4}=8\left[{\left(\lambda_{1}+\lambda_{2}\right)}^{2}+\lambda_{1}\lambda_{2}\right],\;\delta_{3}=4\left(\lambda_{1}+\lambda_{2}\right)\left(2\lambda_{1}^{2}+3\lambda_{1}\lambda_{2}+2\lambda_{2}^{2}\right),\;\\ \delta_{2}=2{\left(\lambda_{1}+\lambda_{2}\right)}^{2}\left(2\lambda_{1}^{2}+\lambda_{1}\lambda_{2}+2\lambda_{2}^{2}\right),\;\delta_{1}=2\lambda_{1}\lambda_{2}{\left(\lambda_{1}+\lambda_{2}\right)}^{3},\delta_{0}=\lambda_{1}^{2}\lambda_{2}^{2}{\left(\lambda_{1}+\lambda_{2}\right)}^{2}. \end{array}$$

**Proof of Proposition 2.**  See Appendix F. □

**Remark 6.**  *When $\lambda_{1}$ or $\lambda_{2}$ is zero, the parallelly-connected network reduces to a serially-connected network with a single path from node 0 to node 3. Thus, in this case, the stationary moments and variance of the age process at node 3 reduce to the corresponding expressions associated with the age process at node 2 in the serially-connected network such that $\lambda_{0}$ and λ are replaced by $\lambda_{s}$ and $\lambda_{1}$ or $\lambda_{2}$. On the other hand, when $\lambda_{1}$ and $\lambda_{2}$ approach ∞, we have $\lim_{\lambda_{1}\to \infty ,\lambda_{2}\to \infty }{\bar{v}}_{\left\{3\right\}}^{\left(1\right)}=\frac{1}{2\lambda_{s}}$, $\lim_{\lambda_{1}\to \infty ,\lambda_{2}\to \infty }{\bar{v}}_{\left\{3\right\}}^{\left(2\right)}=\frac{1}{2\lambda_{s}^{2}}$, and $\lim_{\lambda_{1}\to \infty ,\lambda_{2}\to \infty }\mathrm{var}\left[x_{3}\left(t\right)\right]=\frac{1}{4\lambda_{s}^{2}}$. Note that the stationary moments and variance of $x_{3}\left(t\right)$ reduce to the ones associated with $x_{\left\{1,2\right\}}\left(t\right)$.*

**Remark 7.**  *Note that the stationary moments and variance of the age process at node 3 in (28)–(30) are invariant to exchanging $\lambda_{1}$ and $\lambda_{2}$. Furthermore, for a given $\left(\lambda_{s},\lambda_{2}\right)$, $\left(\lambda_{s},\lambda_{1}\right)$, or $\left(\lambda_{1},\lambda_{2}\right)$, each quantity in (28)–(30) is a monotonically non-increasing function with respect to $\lambda_{1}$, $\lambda_{2}$, or $\lambda_{s}$. This can also be observed in Figure 3.*

**Remark 8.**  *For the same status updating rate from node 0 (i.e., $\lambda_{0}=2\lambda_{s}$) and $\lambda =\lambda_{1}=\lambda_{2}$, one can compare the achievable age performance at node 3 in the parallelly-connected network with the achievable age performance at node 2 in the serially-connected network using Propositions 1 and 2 as follows:*

(32) $$\begin{array}{c} {\bar{v}}_{\left\{2\right\}}^{\left(1\right)}-{\bar{v}}_{\left\{3\right\}}^{\left(1\right)}=\frac{\lambda_{0}}{2\lambda \left(\lambda_{0}+2\lambda \right)}, \end{array}$$

(33) $$\begin{array}{c} {\bar{v}}_{\left\{2\right\}}^{\left(2\right)}-{\bar{v}}_{\left\{3\right\}}^{\left(2\right)}=\frac{3\lambda_{0}^{2}+4\left(\lambda^{2}+2\lambda_{0}\lambda \right)}{2\lambda^{2}{\left(\lambda_{0}+2\lambda \right)}^{2}}, \end{array}$$

(34) $$\begin{array}{c} \mathrm{var}\left[x_{2}\left(t\right)\right]-\mathrm{var}\left[x_{3}\left(t\right)\right]=\frac{3\lambda_{0}\left(\lambda_{0}+4\lambda \right)}{4\lambda^{2}{\left(\lambda_{0}+2\lambda \right)}^{2}}. \end{array}$$

*By inspecting (32)–(34), one can see that these are positive quantities for any choice of values of $\left(\lambda_{0},\lambda \right)$. This certainly indicates that node 3 in the parallelly-connected network achieved a better age performance than the one achievable by node 2 in the serially-connected network. The improvement in the age performance at node 3 resulted from the existence of two status-updating paths from node 0 to node 3, as opposed to only a single path from node 0 to node 2 in the serially-connected network. Furthermore, each quantity in (32)–(34) is a monotonically decreasing function of λ for a given $\lambda_{0}$ such that its value approaches zero as λ→∞. This can also be observed in Figure 3.*

**Remark 9.**  *Due to the symmetry in the configuration of the parallelly-connected network, note that the correlation coefficient between $x_{2}\left(t\right)$ and $x_{3}\left(t\right)$ (i.e., $\mathrm{cor}[x_{2}\left(t\right),x_{3}\left(t\right)]$) can be obtained by replacing $\lambda_{1}$ and $\lambda_{2}$ with $\lambda_{2}$ and $\lambda_{1}$, respectively, in (31). Furthermore, for a given $\left(\lambda_{1},\lambda_{2}\right)$, $\mathrm{cor}[x_{1}\left(t\right),x_{3}\left(t\right)]$ monotonically decreases as a function of $\lambda_{s}$ from $\lim_{\lambda_{s}\to 0}\mathrm{cor}\left[x_{1}\left(t\right),x_{3}\left(t\right)\right]=\frac{1}{2}$ until it approaches $\lim_{\lambda_{s}\to \infty }\mathrm{cor}\left[x_{1}\left(t\right),x_{3}\left(t\right)\right]=0$. On the other hand, for a given $\left(\lambda_{s},\lambda_{2}\right)$, $\mathrm{cor}[x_{1}\left(t\right),x_{3}\left(t\right)]$ monotonically increases as a function of $\lambda_{1}$ from $\lim_{\lambda_{1}\to 0}\mathrm{cor}\left[x_{1}\left(t\right),x_{3}\left(t\right)\right]=0$ until it approaches $\lim_{\lambda_{1}\to \infty }\mathrm{cor}\left[x_{1}\left(t\right),x_{3}\left(t\right)\right]=\frac{4\lambda_{s}^{2}+3\lambda_{s}\lambda_{2}+\lambda_{2}^{2}}{2\left(\lambda_{s}+\lambda_{2}\right)\sqrt{4\lambda_{s}^{2}+2\lambda_{s}\lambda_{2}+\lambda_{2}^{2}}}$. Finally, for a given $\left(\lambda_{s},\lambda_{1}\right)$, one can deduce the following asymptotic results: $\lim_{\lambda_{2}\to 0}\mathrm{cor}\left[x_{1}\left(t\right),x_{3}\left(t\right)\right]=\frac{\lambda_{1}^{2}}{\left(\lambda_{s}+\lambda_{1}\right)\sqrt{\lambda_{s}^{2}+\lambda_{1}^{2}}}$ and $\lim_{\lambda_{2}\to \infty }\mathrm{cor}\left[x_{1}\left(t\right),x_{3}\left(t\right)\right]=\frac{\lambda_{1}\left(\lambda_{s}+\lambda_{1}\right)}{2\left(2\lambda_{s}+\lambda_{1}\right)\sqrt{4\lambda_{s}^{2}+2\lambda_{s}\lambda_{1}+\lambda_{1}^{2}}}$. Clearly, when $\lambda_{2}=0$, there will only be a single status-updating path from node 0 to node 3 (through node 1), and hence we observe that $\mathrm{cor}[x_{1}\left(t\right),x_{3}\left(t\right)]$ reduced to the same expression as $\mathrm{cor}[x_{1}\left(t\right),x_{2}\left(t\right)]$ in (19) for the serially-connected network after replacing $\lambda_{0}$ and λ with $\lambda_{s}$ and $\lambda_{1}$, respectively. Some of the above insights can also be seen in Figure 4.*

### 4.3. Clustered Gossip Networks

**Theorem 5.**  *For the clustered gossip network in Figure 2, the stationary marginal MGFs of the AoI processes at nodes 1, 2, and 3 in the c-th cluster are respectively given by*

(35) $$\begin{array}{c} {\bar{v}}_{\left\{1\right\}}^{\left(n\right)}=\frac{\lambda_{c}}{\lambda_{c}-n}, \end{array}$$

(36) $$\begin{array}{c} {\bar{v}}_{\left\{2\right\}}^{\left(n\right)}=\frac{\lambda_{c}\lambda }{\left(\lambda_{c}-n\right)\left(\lambda -n\right)}, \end{array}$$

(37) $$\begin{array}{c} {\bar{v}}_{\left\{3\right\}}^{\left(n\right)}=\frac{\lambda_{c}\lambda^{2}}{\left(\lambda_{c}-n\right){\left(\lambda -n\right)}^{2}}. \end{array}$$

*Additionally, the stationary joint MGF of each pair of AoI processes at nodes 1, 2, and 3 is given by*

(38) $$\begin{array}{c} {\bar{v}}_{\left\{1\},\{2\right\}}^{\left(n_{1},n_{2}\right)}=\frac{\lambda_{c}\lambda \left[\left(\lambda_{c}+\lambda -n_{2}\right)\left(\lambda_{c}-n_{2}\right)-\lambda_{c}n_{1}\right]}{\left(\lambda_{c}-n_{2}\right)\left(\lambda -n_{2}\right)\left[\lambda_{c}+\lambda -\left(n_{1}+n_{2}\right)\right]\left[\lambda_{c}-\left(n_{1}+n_{2}\right)\right]}, \end{array}$$

(39) $$\begin{array}{c} {\bar{v}}_{\left\{1\},\{3\right\}}^{\left(n_{1},n_{2}\right)}=\frac{\lambda_{c}\lambda^{2}{\left[\lambda_{c}+2\lambda -\left(n_{1}+n_{2}\right)\right]}^{3}\left[\lambda_{c}\left[\lambda_{c}-\left(n_{1}+n_{2}\right)\right]\left[\lambda_{c}+2\lambda -n_{1}-2n_{2}\right]+\left(\lambda_{c}-n_{2}\right){\left(\lambda -n_{2}\right)}^{2}\right]}{\left(\lambda_{c}-n_{2}\right){\left(\lambda -n_{2}\right)}^{2}\left[\lambda_{c}-\left(n_{1}+n_{2}\right)\right]{\left[\lambda_{c}+\lambda -\left(n_{1}+n_{2}\right)\right]}^{2}{\left[\lambda_{c}+2\lambda -\left(n_{1}+n_{2}\right)\right]}^{3}}, \end{array}$$

(40) $$\begin{array}{c} {\bar{v}}_{\left\{2\},\{3\right\}}^{\left(n_{1},n_{2}\right)}=\frac{\lambda_{c}\lambda^{2}\sum_{i=1}^{4}\beta_{i}\left(n_{1},n_{2}\right)}{\left(\lambda_{c}-n_{2}\right){\left(\lambda -n_{2}\right)}^{2}\left[\lambda_{c}-\left(n_{1}+n_{2}\right)\right]\left[\lambda -\left(n_{1}+n_{2}\right)\right]\left[2\lambda -\left(n_{1}+n_{2}\right)\right]{\left[\lambda_{c}+\lambda -\left(n_{1}+n_{2}\right)\right]}^{2}{\left[\lambda_{c}+2\lambda -\left(n_{1}+n_{2}\right)\right]}^{2}}, \end{array}$$

*where*

(41) $$\begin{array}{c} \beta_{1}\left(n_{1},n_{2}\right)=\left(\lambda_{c}-n_{2}\right){\left(\lambda -n_{2}\right)}^{2}{\left[\lambda_{c}+\lambda -\left(n_{1}+n_{2}\right)\right]}^{2}{\left[\lambda_{c}+2\lambda -\left(n_{1}+n_{2}\right)\right]}^{2}, \end{array}$$

(42) $$\begin{array}{c} \beta_{2}\left(n_{1},n_{2}\right)=\lambda^{2}\left(\lambda_{c}-n_{2}\right){\left(\lambda -n_{2}\right)}^{2}\left[\lambda -\left(n_{1}+n_{2}\right)\right]\left[3\lambda_{c}+4\lambda -3\left(n_{1}+n_{2}\right)\right], \end{array}$$

(43) $$\begin{array}{c} \beta_{3}\left(n_{1},n_{2}\right)=\lambda \left(\lambda_{c}-n_{2}\right){\left(\lambda -n_{2}\right)}^{2}\left[\lambda -\left(n_{1}+n_{2}\right)\right]\left[\lambda_{c}-\left(n_{1}+n_{2}\right)\right]\left[\lambda_{c}+\lambda -\left(n_{1}+n_{2}\right)\right], \end{array}$$

(44) $$\begin{array}{cc} \beta_{4}\left(n_{1},n_{2}\right)=\lambda \lambda_{c}\left[\lambda -\left(n_{1}+n_{2}\right)\right]\left[\lambda_{c}-\left(n_{1}+n_{2}\right)\right] & [{\left[\lambda_{c}+2\lambda -\left(n_{1}+n_{2}\right)\right]}^{2}\left[\lambda_{c}+\lambda -\left(n_{1}+n_{2}\right)\right]+\left(\lambda -n_{2}\right){\left[\lambda_{c}+\lambda -\left(n_{1}+n_{2}\right)\right]}^{2}\\ & +\lambda \left(\lambda -n_{2}\right)\left[2\lambda_{c}+3\lambda -2\left(n_{1}+n_{2}\right)\right]]. \end{array}$$

**Proof of Theorem 5.**  See Appendix G. □

**Proposition 3.**  *For the clustered gossip network in Figure 2, the first moment, second moment, and variance of the AoI process at each node in the c-th cluster are given by*

(45) $$\begin{array}{c} {\bar{v}}_{\left\{1\right\}}^{\left(1\right)}=\lambda_{c}^{-1},\;\;{\bar{v}}_{\left\{1\right\}}^{\left(2\right)}=2\lambda_{c}^{-2},\;\;\mathrm{var}\left[x_{1}\left(t\right)\right]=\lambda_{c}^{-2}, \end{array}$$

(46) $$\begin{array}{c} {\bar{v}}_{\left\{2\right\}}^{\left(1\right)}=\lambda_{c}^{-1}+\lambda^{-1},\;\;{\bar{v}}_{\left\{2\right\}}^{\left(2\right)}=2\left(\lambda_{c}^{-2}+\lambda_{c}^{-1}\lambda^{-1}+\lambda^{-2}\right),\;\;\mathrm{var}\left[x_{2}\left(t\right)\right]=\lambda_{c}^{-2}+\lambda^{-2}, \end{array}$$

(47) $$\begin{array}{c} {\bar{v}}_{\left\{3\right\}}^{\left(1\right)}=\lambda_{c}^{-1}+2\lambda^{-1},\;\;{\bar{v}}_{\left\{3\right\}}^{\left(2\right)}=2\left(\lambda_{c}^{-2}+2\lambda_{c}^{-1}\lambda^{-1}+3\lambda^{-2}\right),\;\;\mathrm{var}\left[x_{3}\left(t\right)\right]=\lambda_{c}^{-2}+2\lambda^{-2}. \end{array}$$

*Furthermore, the correlation coefficient between each pair of nodes can be expressed as*

(48) $$\begin{array}{c} \mathrm{cor}\left[x_{1}\left(t\right),x_{2}\left(t\right)\right]=\frac{\lambda^{2}}{\left(\lambda_{c}+\lambda \right)\sqrt{\lambda_{c}^{2}+\lambda^{2}}}, \end{array}$$

(49) $$\begin{array}{c} \mathrm{cor}\left[x_{1}\left(t\right),x_{3}\left(t\right)\right]=\frac{\lambda^{3}}{{\left(\lambda_{c}+\lambda \right)}^{2}\sqrt{2\lambda_{c}^{2}+\lambda^{2}}}, \end{array}$$

(50) $$\begin{array}{c} \mathrm{cor}\left[x_{2}\left(t\right),x_{3}\left(t\right)\right]=\frac{\lambda_{c}^{4}+2\lambda_{c}^{3}\lambda +2\lambda_{c}^{2}\lambda^{2}+2\lambda_{c}\lambda^{3}+2\lambda^{4}}{2{\left(\lambda_{c}+\lambda \right)}^{2}\sqrt{\left(\lambda_{c}^{2}+\lambda^{2}\right)\left(2\lambda_{c}^{2}+\lambda^{2}\right)}}. \end{array}$$

**Proof of Proposition 3.**  See Appendix H. □

**Proposition 4.**  *Let $N_{c}$ denote the set of nodes inside cluster c. For i,j∈{1,2,⋯,C}, the two age processes $x_{N_{i}}\left(t\right)$ and $x_{N_{j}}\left(t\right)$ are not correlated.*

**Proof of Proposition 4.**  See Appendix I. □

**Remark 10.**  *From Proposition 3, one can deduce that ${\bar{v}}_{\left\{1\right\}}^{\left(1\right)}\leq {\bar{v}}_{\left\{2\right\}}^{\left(1\right)}\leq {\bar{v}}_{\left\{3\right\}}^{\left(1\right)}$, ${\bar{v}}_{\left\{1\right\}}^{\left(2\right)}\leq {\bar{v}}_{\left\{2\right\}}^{\left(2\right)}\leq {\bar{v}}_{\left\{3\right\}}^{\left(2\right)}$, and $\mathrm{var}\left[x_{1}\left(t\right)\right]\leq \mathrm{var}\left[x_{2}\left(t\right)\right]\leq \mathrm{var}\left[x_{3}\left(t\right)\right]$ for any choice of values of $\lambda_{c}$ and λ. This follows from the fact that the configuration of each cluster in the clustered gossip network under consideration is a uni-directional ring, where each node has a single status-updating path from node 0 passing through its preceding node in the cluster.*

**Figure 5:**
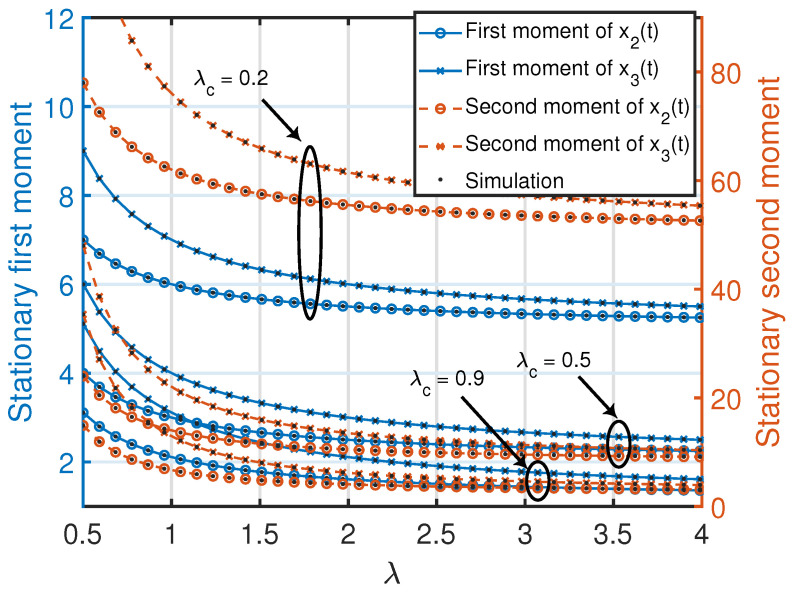
Stationary first and second moments of age processes at the nodes inside the *c*th cluster of the clustered gossip network topology. The simulated curves were obtained from the numerical evaluation of the stationary marginal moments using (10) in Theorem 1.

**Remark 11.**  *Similar to Remark 4, note that the quantities in (46) and (47) associated with the age processes at nodes 2 and 3 are jointly convex functions in $\left(\lambda_{c},\lambda \right)$, where the minimum value (zero) of each function is achieved at $\lambda_{c}=\lambda =\infty$. Furthermore, for a given λ or $\lambda_{c}$, each quantity in (46) and (47) is a monotonically non-increasing function with respect to $\lambda_{c}$ or λ. This can also be observed in Figure 5.*

**Figure 6:**
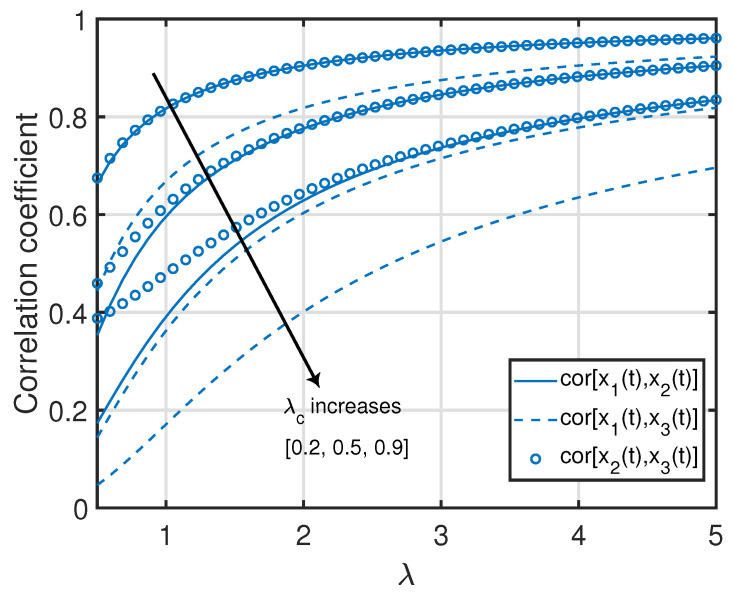
Correlation coefficients between age processes in the clustered gossip network topology.

**Remark 12.**  *Note that the correlation coefficients in (48)–(50) are monotonically non-increasing functions of $\lambda_{c}$ for a given λ, whereas they are monotonically non-decreasing functions of λ for a given $\lambda_{c}$. In particular, $\mathrm{cor}[x_{1}\left(t\right),x_{2}\left(t\right)]$ and $\mathrm{cor}[x_{1}\left(t\right),x_{3}\left(t\right)]$ monotonically increase as functions of λ from $\lim_{\lambda \to 0}\mathrm{cor}\left[x_{1}\left(t\right),x_{2}\left(t\right)\right]=\lim_{\lambda \to 0}\mathrm{cor}\left[x_{1}\left(t\right),x_{3}\left(t\right)\right]=0$ until they approach $\lim_{\lambda \to \infty }\mathrm{cor}\left[x_{1}\left(t\right),x_{2}\left(t\right)\right]=\lim_{\lambda \to \infty }\mathrm{cor}\left[x_{1}\left(t\right),x_{3}\left(t\right)\right]=1$ and monotonically decrease as functions of $\lambda_{c}$ from $\lim_{\lambda_{c}\to 0}\mathrm{cor}\left[x_{1}\left(t\right),x_{2}\left(t\right)\right]=\lim_{\lambda_{c}\to 0}\mathrm{cor}\left[x_{1}\left(t\right),x_{3}\left(t\right)\right]=1$ until they approach $\lim_{\lambda_{c}\to \infty }\mathrm{cor}\left[x_{1}\left(t\right),x_{2}\left(t\right)\right]=\lim_{\lambda_{c}\to \infty }\mathrm{cor}\left[x_{1}\left(t\right),x_{3}\left(t\right)\right]=0$. Additionally, $\mathrm{cor}[x_{2}\left(t\right),x_{3}\left(t\right)]$ monotonically increases as a function of λ from $\lim_{\lambda \to 0}\mathrm{cor}\left[x_{2}\left(t\right),x_{3}\left(t\right)\right]=\frac{1}{2\sqrt{2}}$ until it approaches $\lim_{\lambda \to \infty }\mathrm{cor}\left[x_{2}\left(t\right),x_{3}\left(t\right)\right]=1$ and monotonically decreases as a function of $\lambda_{c}$ from $\lim_{\lambda_{c}\to 0}\mathrm{cor}\left[x_{2}\left(t\right),x_{3}\left(t\right)\right]=1$ until it approaches $\lim_{\lambda_{c}\to \infty }\mathrm{cor}\left[x_{2}\left(t\right),x_{3}\left(t\right)\right]=\frac{1}{2\sqrt{2}}$. These insights can also be seen in Figure 6.*

**Remark 13.**  *Note that the result of Proposition 4 agrees with the intuition. In particular, since the nodes in each cluster are disconnected from the nodes in the other clusters, the two age processes associated with any two arbitrary clusters in the network are uncorrelated.*

**Figure 7:**
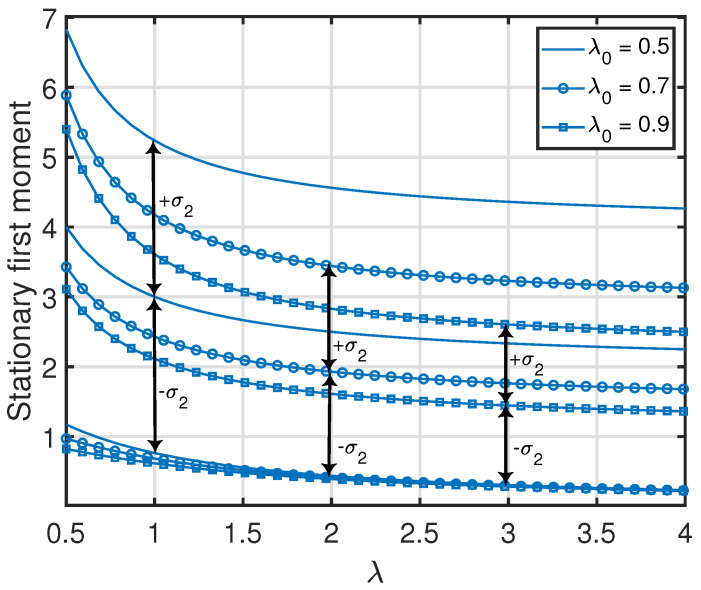
Variance of $x_{2}\left(t\right)$ in the serially-connected network setting. We denote the standard deviation of $x_{2}\left(t\right)$ as $\sigma_{2}$.

**Figure 8:**
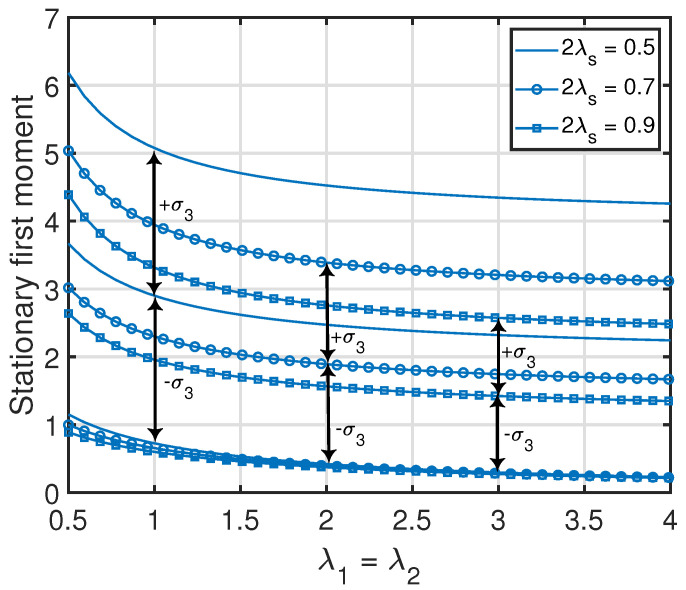
Variance of $x_{3}\left(t\right)$ in the parallelly-connected network setting. We denote the standard deviation of $x_{3}\left(t\right)$ as $\sigma_{3}$.

**Figure 9:**
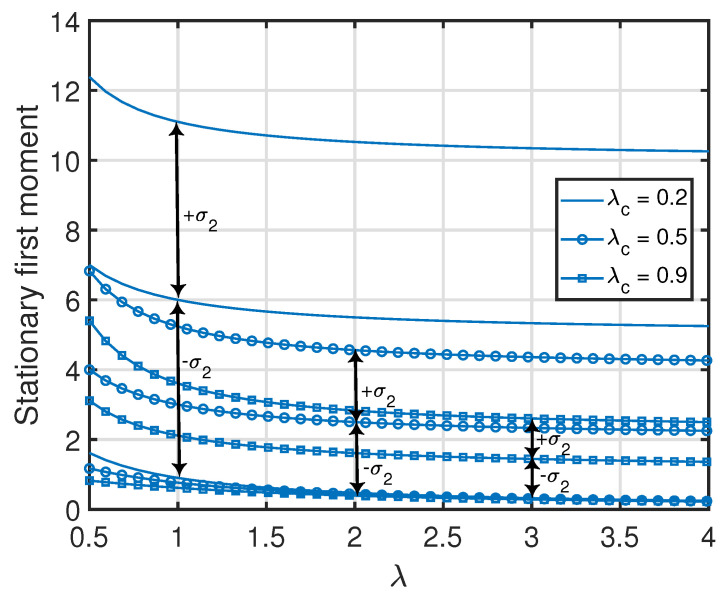
Variance of $x_{2}\left(t\right)$ in the clustered gossip network topology. We denote the standard deviation of $x_{2}\left(t\right)$ as $\sigma_{2}$.

**Figure 10:**
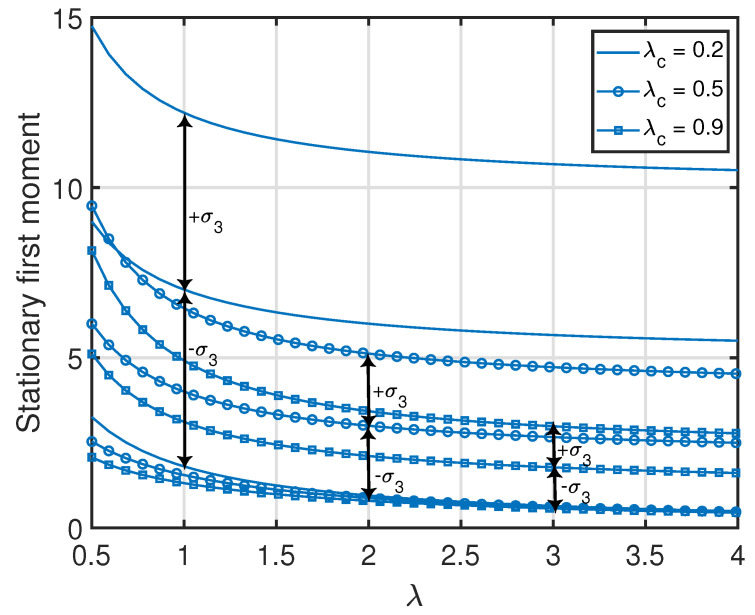
Variance of $x_{3}\left(t\right)$ in the clustered gossip network topology. We denote the standard deviation of $x_{3}\left(t\right)$ as $\sigma_{3}$.

**Remark 14.**  *From Propositions 1–3, one can see that the standard deviation of $x_{1}\left(t\right)$ (i.e., $\sqrt{\mathrm{var}[x_{1}\left(t\right)]}$) was equal to its average value ${\bar{v}}_{\left\{1\right\}}^{\left(1\right)}$. Additionally, the standard deviations of the age processes at the other nodes were relatively large with respect to their average values (which is also demonstrated numerically in Figure 7, Figure 8, Figure 9 and Figure 10). This key insight promotes the importance of incorporating the higher-order moments of age processes in the implementation or optimization of age-aware gossip networks rather than just relying on the average values of the age processes (as has been performed in the existing literature thus far). This insight also demonstrates the need for the development of Theorems 1 and 2 in this paper, which allow the characterization of the marginal or joint MGFs of different age processes in the network that can then be used to evaluate the marginal or joint higher-order moments.*

## 5. Conclusions

In this paper, we developed SHS-based methods that allow the characterization of the stationary marginal and joint MGFs of age processes in a general setting of gossip networks. In particular, we used the SHS framework to derive two systems of first-order ordinary differential equations characterizing the temporal evolution of the marginal and joint MGFs, from which the stationary marginal and joint MGFs were evaluated. Afterward, these methods were applied to derive the stationary marginal and joint MGFs in the following three network topologies: (1) a serially-connected topology, (2) a parallelly-connected topology, and (3) a clustered topology. Using the MGF expressions derived for each network topology, we obtained closed-form expressions for the following quantities: (1) the stationary marginal first and second moments of each age process, (2) the variance of each age process, and (3) the correlation coefficients between all possible pairwise combinations of the age processes. We further characterized the structural properties of these quantities in terms of their convexity and monotonic nature with respect to the status updating rates and provided asymptotic results showing their behaviors when each of the status updating rates became small or large. Our analytical findings demonstrated that the standard deviations of the age processes in each network topology considered in this paper were relatively large with respect to their average values. This key insight promotes the importance of incorporating the higher-order moments of age processes in the implementation and optimization of age-aware gossip networks rather than just relying on the average values of the age processes (as has been performed in the existing literature thus far).

Given the generality of the setting of gossip networks analyzed in this paper, our developed methods can be applied to understand the marginal or joint distributional properties of age processes in any arbitrary gossip network topology. This opens the door for the use of these methods in the future to characterize the stationary marginal or joint moments and MGFs of the age processes in gossip network topologies that have only been analyzed in terms of the stationary first moment of each age process in the network until now. It would also be interesting to investigate how the stationary marginal or joint moments scale as functions of the network size.

## Figures and Tables

**Figure A1:**
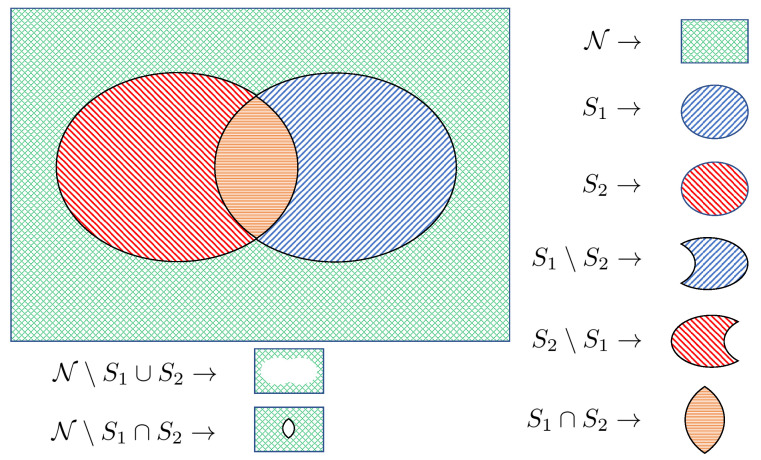
A Venn diagram representation.

## Data Availability

Not applicable.

## Appendix A. Proof of Theorem 1

We derive this result by first using the SHS framework to obtain a system of differential equations characterizing the temporal evolution of the marginal MGFs of the age processes associated with all sets S⊆N. We then obtain the stationary marginal MGFs as the fixed point of this system of equations (i.e., when t→∞). To derive the system of differential equations, we follow a similar approach to that in [15,92], where the idea is to define the test functions $\left\{\psi \left(x\left(t\right)\right)\right\}$ whose expected values $\left\{E\left[\psi \left(x\left(t\right)\right)\right]\right\}$ are quantities of interest. Since we are interested here in the characterization of the marginal MGFs, we define the following class of test functions that is appropriate for this analysis:

(A1) $$\begin{array}{c} \psi_{S}^{\left(n\right)}\left(x\left(t\right)\right)=\exp\left[nx_{S}\left(t\right)\right],\;\forall S\subseteq N, \end{array}$$

where the expected value $E\left[\psi_{S}^{\left(n\right)}\left(x\left(t\right)\right)\right]$ is $v_{S}^{\left(n\right)}\left(t\right)$. We apply the SHS mapping $\psi \left(x\left(t\right)\right)\to L\psi \left(x\left(t\right)\right)$ (known as the extended generator) to every test function in (A1). Since the test functions defined above are time-invariant, it follows from [92] Theorem 1 that the extended generator of a test function $\psi \left(x\left(t\right)\right)$ under the considered piecewise linear SHS is given by

(A2) $$\begin{array}{c} L\psi \left(x\left(t\right)\right)=\frac{d\psi \left(x\left(t\right)\right)}{dx\left(t\right)}1_{N}^{T}+\sum_{l=(i,j)\in L}\lambda_{ij}\left[\psi \left(x^{\prime }\left(t\right)\right)-\psi \left(x\left(t\right)\right)\right], \end{array}$$

where $x^{\prime }\left(t\right)=\left[x_{1}^{\prime }\left(t\right)\;x_{2}^{\prime }\left(t\right)\;\cdots \;x_{N}^{\prime }\left(t\right)\right]$ such that the updated age at node *k*, $x_{k}^{\prime }\left(t\right)$ resulting from the transition (i,j) is given by (1). In addition, note that $x_{S}^{\prime }\left(t\right)=\min_{i\in S}x_{i}^{\prime }\left(t\right)$. Now, we proceed to evaluate $L\psi_{S}^{\left(n\right)}\left(x\left(t\right)\right)$. From the age updating rule in (1), the set of transitions L in (2), and the structure of $\psi_{S}^{\left(n\right)}\left(x\left(t\right)\right)$ in (A1), we have

(A3) $$\begin{array}{c} \frac{d\psi_{S}^{\left(n\right)}\left(x\left(t\right)\right)}{dx\left(t\right)}1_{N}^{T}=n\psi_{S}^{\left(n\right)}\left(x\left(t\right)\right), \end{array}$$

(A4) $$\begin{array}{c} \;\psi_{S}^{\left(n\right)}\left(x^{\prime }\left(t\right)\right)=\exp\left[nx_{S}^{\prime }\left(t\right)\right]=\left\{\begin{array}{cc} \exp\left[n\times 0\right]=1, & l=(0,j),j\in S,\\ \exp\left[nx_{S\cup \{i\}}\left(t\right)\right]=\psi_{S\cup \{i\}}^{\left(n\right)}\left(x\left(t\right)\right), & l=(i,j),j\in S,i\in N\backslash S,\\ \exp\left[nx_{S}\left(t\right)\right]=\psi_{S}^{\left(n\right)}\left(x\left(t\right)\right), & \mathrm{otherwise}. \end{array}\right. \end{array}$$

Substituting (A3) and (A4) into (A2) gives

(A5) $$\begin{array}{c} \;L\psi_{S}^{\left(n\right)}\left(x\left(t\right)\right)=n\psi_{S}^{\left(n\right)}\left(x\left(t\right)\right)+\sum_{j\in S}\lambda_{0j}\left[1-\psi_{S}^{\left(n\right)}\left(x\left(t\right)\right)\right]+\sum_{i\in N\backslash S}\sum_{j\in S}\lambda_{ij}\left[\psi_{S\cup \{i\}}^{\left(n\right)}\left(x\left(t\right)\right)-\psi_{S}^{\left(n\right)}\left(x\left(t\right)\right)\right], \end{array}$$

The system of differential equations characterizing the temporal evolution of the marginal MGFs ${\left\{v_{S}^{\left(n\right)}\left(t\right)\right\}}_{S\subseteq N}$ can be derived by applying Dynkin’s formula [92] to each test function and its associated extended generator. In particular, for a test function $\psi \left(x\left(t\right)\right)$, the Dynkin’s formula can be expressed as

(A6) $$\begin{array}{c} \frac{dE[\psi \left(x\left(t\right)\right)]}{dt}=E\left[L\psi \left(x\left(t\right)\right)\right]. \end{array}$$

Plugging $\psi_{S}^{\left(n\right)}\left(x\left(t\right)\right)$ and $L\psi_{S}^{\left(n\right)}\left(x\left(t\right)\right)$ into (A6) gives

(A7) $$\begin{array}{cc} {\dot{v}}_{S}^{\left(n\right)}\left(t\right) & =nv_{S}^{\left(n\right)}\left(t\right)+\sum_{j\in S}\lambda_{0j}\left[1-v_{S}^{\left(n\right)}\left(t\right)\right]+\sum_{i\in N\backslash S}\sum_{j\in S}\lambda_{ij}\left[v_{S\cup \{i\}}^{\left(n\right)}\left(t\right)-v_{S}^{\left(n\right)}\left(t\right)\right]\\ \; & \overset{\left(a\right)}{=}\lambda_{0}\left(S\right)+v_{S}^{\left(n\right)}\left(t\right)\left[n-\lambda_{0}\left(S\right)-\sum_{i\in N(S)}\lambda_{i}\left(S\right)\right]+\sum_{i\in N(S)}\lambda_{i}\left(S\right)v_{S\cup \{i\}}^{\left(n\right)}\left(t\right), \end{array}$$

where step (a) directly follows from the definitions of $\lambda_{i}\left(S\right)$ and N(S) in (7) and (8), respectively. Note that there exists a range of *n* values for which the differential equation in (A7) is asymptotically stable for any arbitrary set S⊆N. To see this, let us first express ${\dot{v}}_{N}^{\left(n\right)}\left(t\right)$ using (A7) as follows:

(A8) $$\begin{array}{c} {\dot{v}}_{N}^{\left(n\right)}\left(t\right)=\lambda_{0}\left(N\right)+v_{N}^{\left(n\right)}\left(t\right)\left[n-\lambda_{0}\left(N\right)\right]. \end{array}$$

For $0\leq n<\lambda_{0}\left(N\right)$, (A8) is asymptotically stable, and the stationary marginal MGF ${\bar{v}}_{N}^{\left(n\right)}$ can be obtained by setting ${\dot{v}}_{N}^{\left(n\right)}\left(t\right)$ to zero and replacing $v_{N}^{\left(n\right)}\left(t\right)$ with ${\bar{v}}_{N}^{\left(n\right)}$. Now, when S=N\{k}, (A7) is given by

(A9) $$\begin{array}{c} {\dot{v}}_{S}^{\left(n\right)}\left(t\right)=\lambda_{0}\left(S\right)+v_{S}^{\left(n\right)}\left(t\right)\left[n-\lambda_{0}\left(S\right)-\lambda_{k}\left(S\right)\right]+\lambda_{k}\left(S\right)v_{N}^{\left(n\right)}\left(t\right). \end{array}$$

For $0\leq n<\min\left[\lambda_{0}\left(S\right)+\lambda_{k}\left(S\right),\lambda_{0}\left(N\right)\right]$ and S=N\{k}, $v_{N}^{\left(n\right)}\left(t\right)$ converges as t→∞, and the differential equation in (A9) is asymptotically stable. The stationary marginal MGF ${\bar{v}}_{N\backslash \{k\}}^{\left(n\right)}$ is then the fixed point of (A9), which can be obtained after setting the derivative to zero. Afterward, when $S=N\backslash \{k_{1},k_{2}\}$, one can follow the above procedure to obtain the range of *n* values under which (A7) is asymptotically stable. Generally, for an arbitrary set *S*, there exists a threshold δ such that for n∈[0,δ), the stationary marginal MGF ${\bar{v}}_{S}^{\left(n\right)}$ is the fixed point of (A7).

Finally, the stationary marginal *m*th moment ${\bar{v}}_{S}^{\left(m\right)}$ in (10) can be obtained by substituting ψ(x(t)) in (A2) with $\psi_{S}^{\left(m\right)}\left(x\left(t\right)\right)=x_{S}^{m}\left(t\right)$ and following similar steps to those in (A2)–(A9). This completes the proof.

## Appendix B. Proof of Theorem 2

The flow of this proof is similar to that for Theorem 1 in Appendix A. In particular, we start by constructing a class of test functions that is appropriate for the joint MGF analysis. We then use (A2) to derive the extended generator for each test function, which is then plugged into Dynkin’s formula in (A6) to obtain the system of differential equations characterizing the temporal evolution of the joint MGFs ${\left\{v_{S_{1},S_{2}}^{\left(n_{1},n_{2}\right)}\right\}}_{S_{1},S_{2}\subseteq N}$. The class of test functions we define here for the joint MGF analysis is given by

(A10) $$\begin{array}{c} \psi_{S_{1},S_{2}}^{\left(n_{1},n_{2}\right)}\left(x\left(t\right)\right)=\exp\left[n_{1}x_{S_{1}}\left(t\right)+n_{2}x_{S_{2}}\left(t\right)\right],\;\forall S_{1},S_{2}\subseteq N, \end{array}$$

such that the expected value $E\left[\psi_{S_{1},S_{2}}^{\left(n_{1},n_{2}\right)}\left(x\left(t\right)\right)\right]$ is $v_{S_{1},S_{2}}^{\left(n_{1},n_{2}\right)}\left(t\right)$. For such a structure of test functions, we have

(A11) $$\begin{array}{c} \frac{d\psi_{S_{1},S_{2}}^{\left(n_{1},n_{2}\right)}\left(x\left(t\right)\right)}{dx\left(t\right)}1_{N}^{T}=\left(n_{1}+n_{2}\right)\psi_{S_{1},S_{2}}^{\left(n_{1},n_{2}\right)}\left(x\left(t\right)\right). \end{array}$$

Compared with the proof for Theorem 1 in Appendix A, a key challenge in the derivation of the extended generator here is to carefully identify all the possible transitions in L that result in having $\psi_{S_{1},S_{2}}^{\left(n_{1},n_{2}\right)}\left(x^{\prime }\left(t\right)\right)\neq \psi_{S_{1},S_{2}}^{\left(n_{1},n_{2}\right)}\left(x\left(t\right)\right)$. We provide Figure A1 to help one easily visualize the following arguments. For the first subset of transitions {(0,j):j∈N}⊂L, we have

(A12) $$\begin{array}{c} \psi_{S_{1},S_{2}}^{\left(n_{1},n_{2}\right)}\left(x^{\prime }\left(t\right)\right)=\exp\left[n_{1}x_{S_{1}}^{\prime }\left(t\right)+n_{2}x_{S_{2}}^{\prime }\left(t\right)\right]=\left\{\begin{array}{cc} \psi_{S_{2}}^{\left(n_{2}\right)}\left(x\left(t\right)\right), & l=\left(0,j\right),j\in S_{1}\backslash S_{2},\\ \psi_{S_{1}}^{\left(n_{1}\right)}\left(x\left(t\right)\right), & l=\left(0,j\right),j\in S_{2}\backslash S_{1},\\ 1, & l=\left(0,j\right),j\in S_{1}\cap S_{2},\\ \psi_{S_{1},S_{2}}^{\left(n_{1},n_{2}\right)}\left(x\left(t\right)\right), & \mathrm{otherwise}. \end{array}\right. \end{array}$$

To help one easily grasp the different cases in (A12), we elaborate more on the construction of the first case, and the other cases can be interpreted similarly. In particular, when $j\in S_{1}\backslash S_{2}$, the transition (0,j) results in resetting the age of $S_{1}$ to zero, whereas the age of $S_{2}$ will not change. As a result, $\psi_{S_{1},S_{2}}^{\left(n_{1},n_{2}\right)}\left(x^{\prime }\left(t\right)\right)=\exp\left[n_{1}\times 0+n_{2}\times x_{S_{2}}\left(t\right)\right]\overset{\left(a\right)}{=}\psi_{S_{2}}^{\left(n_{2}\right)}\left(x\left(t\right)\right)$, where step (a) follows from (A1).

For the second subset of transitions {(i,j):i,j∈N}⊂L, we have

(A13) $$\begin{array}{c} \psi_{S_{1},S_{2}}^{\left(n_{1},n_{2}\right)}\left(x^{\prime }\left(t\right)\right)=\exp\left[n_{1}x_{S_{1}}^{\prime }\left(t\right)+n_{2}x_{S_{2}}^{\prime }\left(t\right)\right]=\left\{\begin{array}{cc} \psi_{S_{1}\cup \left\{i\right\},S_{2}}^{\left(n_{1},n_{2}\right)}\left(x\left(t\right)\right), & l=\left(i,j\right),j\in S_{1}\backslash S_{2},i\in N\backslash S_{1},\\ \psi_{S_{1},S_{2}\cup \left\{i\right\}}^{\left(n_{1},n_{2}\right)}\left(x\left(t\right)\right), & l=\left(i,j\right),j\in S_{2}\backslash S_{1},i\in N\backslash S_{2},\\ \psi_{S_{1}\cup \left\{i\right\},S_{2}\cup \left\{i\right\}}^{\left(n_{1},n_{2}\right)}\left(x\left(t\right)\right), & l=\left(i,j\right),j\in S_{1}\cap S_{2},i\in N\backslash S_{1}\cup S_{2},\\ \psi_{S_{1},S_{2}\cup \left\{i\right\}}^{\left(n_{1},n_{2}\right)}\left(x\left(t\right)\right), & l=\left(i,j\right),j\in S_{1}\cap S_{2},i\in S_{1}\backslash S_{2},\\ \psi_{S_{1}\cup \left\{i\right\},S_{2}}^{\left(n_{1},n_{2}\right)}\left(x\left(t\right)\right), & l=\left(i,j\right),j\in S_{1}\cap S_{2},i\in S_{2}\backslash S_{1},\\ \psi_{S_{1},S_{2}}^{\left(n_{1},n_{2}\right)}\left(x\left(t\right)\right), & \mathrm{otherwise}. \end{array}\right. \end{array}$$

Plugging (A11)–(A13) into (A2) gives

(A14) $$\begin{array}{cc} \;L\psi_{S_{1},S_{2}}^{\left(n_{1},n_{2}\right)} & \left(x\left(t\right)\right)=\left(n_{1}+n_{2}\right)\psi_{S_{1},S_{2}}^{\left(n_{1},n_{2}\right)}\left(x\left(t\right)\right)+\sum_{j\in S_{1}\backslash S_{2}}\lambda_{0j}\left[\psi_{S_{2}}^{\left(n_{2}\right)}\left(x\left(t\right)\right)-\psi_{S_{1},S_{2}}^{\left(n_{1},n_{2}\right)}\left(x\left(t\right)\right)\right]+\sum_{j\in S_{2}\backslash S_{1}}\lambda_{0j}\left[\psi_{S_{1}}^{\left(n_{1}\right)}\left(x\left(t\right)\right)-\psi_{S_{1},S_{2}}^{\left(n_{1},n_{2}\right)}\left(x\left(t\right)\right)\right]\\ & +\sum_{j\in S_{1}\cap S_{2}}\lambda_{0j}\left[1-\psi_{S_{1},S_{2}}^{\left(n_{1},n_{2}\right)}\left(x\left(t\right)\right)\right]+\sum_{i\in N\backslash S_{1}}\sum_{j\in S_{1}\backslash S_{2}}\lambda_{ij}\left[\psi_{S_{1}\cup \left\{i\right\},S_{2}}^{\left(n_{1},n_{2}\right)}\left(x\left(t\right)\right)-\psi_{S_{1},S_{2}}^{\left(n_{1},n_{2}\right)}\left(x\left(t\right)\right)\right]\\ & +\sum_{i\in N\backslash S_{2}}\sum_{j\in S_{2}\backslash S_{1}}\lambda_{ij}\left[\psi_{S_{1},S_{2}\cup \left\{i\right\}}^{\left(n_{1},n_{2}\right)}\left(x\left(t\right)\right)-\psi_{S_{1},S_{2}}^{\left(n_{1},n_{2}\right)}\left(x\left(t\right)\right)\right]+\sum_{i\in N\backslash S_{1}\cup S_{2}}\sum_{j\in S_{1}\cap S_{1}}\lambda_{ij}\left[\psi_{S_{1}\cup \left\{i\right\},S_{2}\cup \left\{i\right\}}^{\left(n_{1},n_{2}\right)}\left(x\left(t\right)\right)-\psi_{S_{1},S_{2}}^{\left(n_{1},n_{2}\right)}\left(x\left(t\right)\right)\right]\\ & +\sum_{i\in S_{1}\backslash S_{2}}\sum_{j\in S_{1}\cap S_{2}}\lambda_{ij}\left[\psi_{S_{1},S_{2}\cup \left\{i\right\}}^{\left(n_{1},n_{2}\right)}\left(x\left(t\right)\right)-\psi_{S_{1},S_{2}}^{\left(n_{1},n_{2}\right)}\left(x\left(t\right)\right)\right]+\sum_{i\in S_{2}\backslash S_{1}}\sum_{j\in S_{1}\cap S_{2}}\lambda_{ij}\left[\psi_{S_{1}\cup \left\{i\right\},S_{2}}^{\left(n_{1},n_{2}\right)}\left(x\left(t\right)\right)-\psi_{S_{1},S_{2}}^{\left(n_{1},n_{2}\right)}\left(x\left(t\right)\right)\right]. \end{array}$$

By applying Dynkin’s formula in (A6) to $\psi_{S_{1},S_{2}}^{\left(n_{1},n_{2}\right)}\left(x\left(t\right)\right)$ and $L\psi_{S_{1},S_{2}}^{\left(n_{1},n_{2}\right)}\left(x\left(t\right)\right)$, we have

(A15) $$\begin{array}{cc} \;{\dot{v}}_{S_{1},S_{2}}^{\left(n_{1},n_{2}\right)}\left(t\right) & =\left(n_{1}+n_{2}\right)v_{S_{1},S_{2}}^{\left(n_{1},n_{2}\right)}\left(t\right)+\sum_{j\in S_{1}\backslash S_{2}}\lambda_{0j}\left[v_{S_{2}}^{\left(n_{2}\right)}\;\left(t\right)-v_{S_{1},S_{2}}^{\left(n_{1},n_{2}\right)}\left(t\right)\right]+\sum_{j\in S_{2}\backslash S_{1}}\lambda_{0j}\left[v_{S_{1}}^{\left(n_{1}\right)}\left(t\right)-v_{S_{1},S_{2}}^{\left(n_{1},n_{2}\right)}\left(t\right)\right]\\ & +\sum_{j\in S_{1}\cap S_{2}}\lambda_{0j}\left[1-v_{S_{1},S_{2}}^{\left(n_{1},n_{2}\right)}\left(t\right)\right]+\sum_{i\in N\backslash S_{1}}\sum_{j\in S_{1}\backslash S_{2}}\lambda_{ij}\left[v_{S_{1}\cup \left\{i\right\},S_{2}}^{\left(n_{1},n_{2}\right)}\left(t\right)-v_{S_{1},S_{2}}^{\left(n_{1},n_{2}\right)}\left(t\right)\right]\\ & +\sum_{i\in N\backslash S_{2}}\sum_{j\in S_{2}\backslash S_{1}}\lambda_{ij}\left[v_{S_{1},S_{2}\cup \left\{i\right\}}^{\left(n_{1},n_{2}\right)}\left(t\right)-v_{S_{1},S_{2}}^{\left(n_{1},n_{2}\right)}\left(t\right)\right]+\sum_{i\in N\backslash S_{1}\cup S_{2}}\sum_{j\in S_{1}\cap S_{1}}\lambda_{ij}\left[v_{S_{1}\cup \left\{i\right\},S_{2}\cup \left\{i\right\}}^{\left(n_{1},n_{2}\right)}\left(t\right)-v_{S_{1},S_{2}}^{\left(n_{1},n_{2}\right)}\left(t\right)\right]\\ & +\sum_{i\in S_{1}\backslash S_{2}}\sum_{j\in S_{1}\cap S_{2}}\lambda_{ij}\left[v_{S_{1},S_{2}\cup \left\{i\right\}}^{\left(n_{1},n_{2}\right)}\left(t\right)-v_{S_{1},S_{2}}^{\left(n_{1},n_{2}\right)}\left(t\right)\right]+\sum_{i\in S_{2}\backslash S_{1}}\sum_{j\in S_{1}\cap S_{2}}\lambda_{ij}\left[v_{S_{1}\cup \left\{i\right\},S_{2}}^{\left(n_{1},n_{2}\right)}\left(t\right)-v_{S_{1},S_{2}}^{\left(n_{1},n_{2}\right)}\left(t\right)\right]\\ & \overset{\left(a\right)}{=}v_{S_{1},S_{2}}^{\left(n_{1},n_{2}\right)}\left(t\right)\left[\left(n_{1}+n_{2}\right)-\lambda_{0}\left(S_{1}\cup S_{2}\right)-\sum_{i\in N\backslash (S_{1}\cap S_{2})}\lambda_{i}\left(S_{1}\cap S_{2}\right)-\sum_{i\in N\backslash S_{1}}\lambda_{i}\left(S_{1}\backslash S_{2}\right)-\sum_{i\in N\backslash S_{2}}\lambda_{i}\left(S_{2}\backslash S_{1}\right)\right]\\ & +\lambda_{0}\left(S_{1}\cap S_{2}\right)+\lambda_{0}\left(S_{1}\backslash S_{2}\right)v_{S_{2}}^{\left(n_{2}\right)}\left(t\right)+\lambda_{0}\left(S_{2}\backslash S_{1}\right)v_{S_{1}}^{\left(n_{1}\right)}\left(t\right)+\sum_{i\in N\backslash S_{1}}\lambda_{i}\left(S_{1}\backslash S_{2}\right)v_{S_{1}\cup \left\{i\right\},S_{2}}^{\left(n_{1},n_{2}\right)}\left(t\right)\\ & +\sum_{i\in N\backslash S_{2}}\lambda_{i}\left(S_{2}\backslash S_{1}\right)v_{S_{1},S_{2}\cup \left\{i\right\}}^{\left(n_{1},n_{2}\right)}\left(t\right)+\sum_{i\in N\backslash (S_{1}\cup S_{2})}\lambda_{i}\left(S_{1}\cap S_{2}\right)v_{S_{1}\cup \left\{i\right\},S_{2}\cup \left\{i\right\}}^{\left(n_{1},n_{2}\right)}\left(t\right)+\sum_{i\in S_{1}\backslash S_{2}}\lambda_{i}\left(S_{1}\cap S_{2}\right)v_{S_{1},S_{2}\cup \left\{i\right\}}^{\left(n_{1},n_{2}\right)}\left(t\right)\\ & +\sum_{i\in S_{2}\backslash S_{1}}\lambda_{i}\left(S_{1}\cap S_{2}\right)v_{S_{1}\cup \left\{i\right\},S_{2}}^{\left(n_{1},n_{2}\right)}\left(t\right), \end{array}$$

where step (a) follows from applying the definition of $\lambda_{i}\left(S\right)$ in (7), followed by some algebraic simplifications. Now, following a similar procedure to that in (A8) and (A9) in Appendix A, one can show that for any two arbitrary sets $S_{1}$ and $S_{2}$, there exists a threshold δ (such that $0\leq n_{1}+n_{2}<\delta$) under which the differential equation in (A15) is asymptotically stable. Thus, the final expression of the stationary joint MGF ${\bar{v}}_{S_{1},S_{2}}^{\left(n_{1},n_{2}\right)}$ in (11) can be obtained by taking the limit as t→∞ in (A15) (i.e., setting ${\dot{v}}_{S_{1},S_{2}}^{\left(n_{1},n_{2}\right)}\left(t\right)$ to zero and replacing $v_{S_{1},S_{2}}^{\left(n_{1},n_{2}\right)}\left(t\right)$ with ${\bar{v}}_{S_{1},S_{2}}^{\left(n_{1},n_{2}\right)}$).

Finally, the stationary joint $\left(m_{1},m_{2}\right)$th moment ${\bar{v}}_{S_{1},S_{2}}^{\left(m_{1},m_{2}\right)}$ in (12) can be obtained by substituting ψ(x(t)) in (A2) with $\psi_{S_{1},S_{2}}^{\left(m_{1},m_{2}\right)}\left(x\left(t\right)\right)=x_{S_{1}}^{m_{1}}\left(t\right)x_{S_{2}}^{m_{2}}\left(t\right)$ and following similar steps to those in (A11)–(A15). This completes the proof.

## Appendix C. Proof of Theorem 3

We start the proof by showing how one can use Theorem 1 to obtain the stationary marginal MGF of the AoI or age process at each node in the network. In particular, by observing the set of transitions in Figure 1a, repeated application of (9) gives

(A16) $$\begin{array}{c} {\bar{v}}_{\left\{1\right\}}^{\left(n\right)}=\frac{\lambda_{0}}{\lambda_{0}-n}, \end{array}$$

(A17) $$\begin{array}{c} {\bar{v}}_{\left\{2\right\}}^{\left(n\right)}=\frac{\lambda {\bar{v}}_{\left\{1,2\right\}}^{\left(n\right)}}{\lambda -n}, \end{array}$$

(A18) $$\begin{array}{c} {\bar{v}}_{\left\{1,2\right\}}^{\left(n\right)}=\frac{\lambda_{0}}{\lambda_{0}-n}. \end{array}$$

By substituting (A18) into (A17), we obtain

(A19) $$\begin{array}{c} {\bar{v}}_{\left\{2\right\}}^{\left(n\right)}=\frac{\lambda_{0}\lambda }{\left(\lambda_{0}-n\right)\left(\lambda -n\right)}. \end{array}$$

Now, we proceed to the evaluation of the stationary joint MGF ${\bar{v}}_{\left\{2\},\{1\right\}}^{\left(n_{1},n_{2}\right)}$ using Theorem 2. In particular, by applying (11) twice (the first time for $S_{1}=\left\{2\right\}$ and $S_{2}=\left\{1\right\}$ and the second time for $S_{1}=\left\{1,2\right\}$ and $S_{2}=\left\{1\right\}$), we obtain

(A20) $$\begin{array}{c} {\bar{v}}_{\left\{2\},\{1\right\}}^{\left(n_{1},n_{2}\right)}\left[\lambda_{0}+\lambda -\left(n_{1}+n_{2}\right)\right]=\lambda_{0}{\bar{v}}_{\left\{2\right\}}^{\left(n_{1}\right)}+\lambda {\bar{v}}_{\left\{1,2\},\{1\right\}}^{\left(n_{1},n_{2}\right)}, \end{array}$$

(A21) $$\begin{array}{c} {\bar{v}}_{\left\{1,2\},\{1\right\}}^{\left(n_{1},n_{2}\right)}\left[\lambda_{0}-\left(n_{1}+n_{2}\right)\right]=\lambda_{0}. \end{array}$$

The final expression of ${\bar{v}}_{\left\{2\},\{1\right\}}^{\left(n_{1},n_{2}\right)}$ in (16) can be obtained by substituting ${\bar{v}}_{\left\{2\right\}}^{\left(n_{1}\right)}$ and ${\bar{v}}_{\left\{1,2\},\{1\right\}}^{\left(n_{1},n_{2}\right)}$ from (A17) and (A21), respectively, into (A20).

## Appendix D. Proof of Proposition 1

The stationary marginal *m*th moment of the age process at node i∈N={1,2} (i.e., ${\bar{v}}_{\left\{i\right\}}^{\left(m\right)}$) is given by

(A22) $$\begin{array}{c} {\bar{v}}_{\left\{i\right\}}^{\left(m\right)}=\frac{d^{m}\left[{\bar{v}}_{\left\{i\right\}}^{\left(n\right)}\right]}{dn^{m}}{|}_{n=0}. \end{array}$$

Furthermore, for i,j∈N, the stationary joint moment ${\bar{v}}_{\left\{i\},\{j\right\}}^{\left(m_{1},m_{2}\right)}$ of the two age processes at nodes *i* and *j* is given by

(A23) $$\begin{array}{c} {\bar{v}}_{\left\{i\},\{j\right\}}^{\left(m_{1},m_{2}\right)}=\frac{\partial^{m_{1}+m_{2}}\left[{\bar{v}}_{\left\{i\},\{j\right\}}^{\left(n_{1},n_{2}\right)}\right]}{\partial n_{1}^{m_{1}}\partial n_{2}^{m_{2}}}{|}_{n_{1}=0,n_{2}=0}. \end{array}$$

The marginal first and second moments of the age process at each node in the serially-connected network (in (A22) and (A23)) can be obtained by plugging the marginal MGF expressions derived in Theorem 3 into (A22). Furthermore, the variance of the age process at node *i* is given by

(A24) $$\begin{array}{c} \mathrm{var}\left[x_{i}\left(t\right)\right]={\bar{v}}_{\left\{i\right\}}^{\left(2\right)}-{\left({\bar{v}}_{\left\{i\right\}}^{\left(1\right)}\right)}^{2}. \end{array}$$

Finally, for nodes i,j∈N, the correlation coefficient can be evaluated as follows:

(A25) $$\begin{array}{c} \mathrm{cor}\left[x_{i}\left(t\right),x_{j}\left(t\right)\right]=\frac{{\bar{v}}_{\left\{i\},\{j\right\}}^{\left(1,1\right)}-{\bar{v}}_{\left\{i\right\}}^{\left(1\right)}{\bar{v}}_{\left\{j\right\}}^{\left(1\right)}}{\sqrt{\mathrm{var}\left[x_{i}\left(t\right)\right]}\sqrt{\mathrm{var}\left[x_{j}\left(t\right)\right]}}, \end{array}$$

In order to obtain $\mathrm{cor}\left[x_{1}\left(t\right),x_{2}\left(t\right)\right]$, what remains is only to evaluate ${\bar{v}}_{\left\{2\},\{1\right\}}^{\left(1,1\right)}$ from (A23) (using the joint MGF expression in (16)) as

(A26) $$\begin{array}{c} {\bar{v}}_{\left\{2\},\{1\right\}}^{\left(1,1\right)}=\frac{\lambda_{0}^{2}+2\lambda_{0}\lambda +2\lambda^{2}}{\lambda \lambda_{0}^{2}\left(\lambda_{0}+\lambda \right)}. \end{array}$$

By noting that

(A27) $$\begin{array}{c} {\bar{v}}_{\left\{2\},\{1\right\}}^{\left(1,1\right)}-{\bar{v}}_{\left\{2\right\}}^{\left(1\right)}{\bar{v}}_{\left\{1\right\}}^{\left(1\right)}=\frac{\lambda_{0}^{2}+2\lambda_{0}\lambda +2\lambda^{2}}{\lambda \lambda_{0}^{2}\left(\lambda_{0}+\lambda \right)}-\frac{\lambda_{0}+\lambda }{\lambda_{0}^{2}\lambda }=\frac{\lambda }{\lambda_{0}^{2}\left(\lambda_{0}+\lambda \right)}, \end{array}$$

then the final expression of $\mathrm{cor}[x_{1}\left(t\right),x_{2}\left(t\right)]$ in (19) can be obtained, which completes the proof.

## Appendix E. Proof of Theorem 4

For the parallelly-connected network in Figure 1b, the stationary marginal MGF of the age process at each node i∈N={1,2,3} can be derived by repeatedly applying (9) as follows:

(A28) $$\begin{array}{c} {\bar{v}}_{\left\{3\right\}}^{\left(n\right)}=\frac{\lambda_{1}{\bar{v}}_{\left\{1,3\right\}}^{\left(n\right)}+\lambda_{2}{\bar{v}}_{\left\{2,3\right\}}^{\left(n\right)}}{\lambda_{1}+\lambda_{2}-n}, \end{array}$$

(A29) $$\begin{array}{c} {\bar{v}}_{\left\{1,3\right\}}^{\left(n\right)}=\frac{\lambda_{s}+\lambda_{2}{\bar{v}}_{\left\{1,2,3\right\}}^{\left(n\right)}}{\lambda_{s}+\lambda_{2}-n}, \end{array}$$

(A30) $$\begin{array}{c} {\bar{v}}_{\left\{2,3\right\}}^{\left(n\right)}=\frac{\lambda_{s}+\lambda_{1}{\bar{v}}_{\left\{1,2,3\right\}}^{\left(n\right)}}{\lambda_{s}+\lambda_{1}-n}, \end{array}$$

(A31) $$\begin{array}{c} {\bar{v}}_{\left\{1,2,3\right\}}^{\left(n\right)}=\frac{2\lambda_{s}}{2\lambda_{s}-n}, \end{array}$$

(A32) $$\begin{array}{c} {\bar{v}}_{\left\{1\right\}}^{\left(n\right)}={\bar{v}}_{\left\{2\right\}}^{\left(n\right)}=\frac{\lambda_{s}}{\lambda_{s}-n},. \end{array}$$

The final expression of ${\bar{v}}_{\left\{3\right\}}^{\left(n\right)}$ in (21) can be obtained by substituting ${\bar{v}}_{\left\{1,3\right\}}^{\left(n\right)}$ and ${\bar{v}}_{\left\{2,3\right\}}^{\left(n\right)}$ from (A29)–(A31) into (A28).

We now derive the stationary joint MGF ${\bar{v}}_{\left\{3\},\{1\right\}}^{\left(n_{1},n_{2}\right)}$ by repeatedly applying (11) as follows:

(A33) $$\begin{array}{c} {\bar{v}}_{\left\{3\},\{1\right\}}^{\left(n_{1},n_{2}\right)}\left[\lambda_{s}+\lambda_{1}+\lambda_{2}-\left(n_{1}+n_{2}\right)\right]=\lambda_{s}{\bar{v}}_{\left\{3\right\}}^{\left(n_{1}\right)}+\lambda_{1}{\bar{v}}_{\left\{1,3\},\{1\right\}}^{\left(n_{1},n_{2}\right)}+\lambda_{2}{\bar{v}}_{\left\{2,3\},\{1\right\}}^{\left(n_{1},n_{2}\right)}, \end{array}$$

(A34) $$\begin{array}{c} {\bar{v}}_{\left\{1,3\},\{1\right\}}^{\left(n_{1},n_{2}\right)}\left[\lambda_{s}+\lambda_{2}-\left(n_{1}+n_{2}\right)\right]=\lambda_{s}+\lambda_{2}{\bar{v}}_{\left\{1,2,3\},\{1\right\}}^{\left(n_{1},n_{2}\right)}, \end{array}$$

(A35) $$\begin{array}{c} {\bar{v}}_{\left\{2,3\},\{1\right\}}^{\left(n_{1},n_{2}\right)}\left[2\lambda_{s}+\lambda_{1}-\left(n_{1}+n_{2}\right)\right]=\lambda_{s}\left({\bar{v}}_{\left\{1\right\}}^{\left(n_{2}\right)}+{\bar{v}}_{\left\{2,3\right\}}^{\left(n_{1}\right)}\right)+\lambda_{1}{\bar{v}}_{\left\{1,2,3\},\{1\right\}}^{\left(n_{1},n_{2}\right)}, \end{array}$$

(A36) $$\begin{array}{c} {\bar{v}}_{\left\{1,2,3\},\{1\right\}}^{\left(n_{1},n_{2}\right)}\left[2\lambda_{s}-\left(n_{1}+n_{2}\right)\right]=\lambda_{s}+\lambda_{s}{\bar{v}}_{\left\{1\right\}}^{\left(n_{2}\right)}. \end{array}$$

By substituting (A34)–(A36) into (A33), ${\bar{v}}_{\left\{3\},\{1\right\}}^{\left(n_{1},n_{2}\right)}$ can expressed as

(A37) $$\begin{array}{cc} {\bar{v}}_{\left\{3\},\{1\right\}}^{\left(n_{1},n_{2}\right)}= & \frac{1}{\left[\lambda_{s}+\lambda_{1}+\lambda_{2}-\left(n_{1}+n_{2}\right)\right]\left[2\lambda_{s}+\lambda_{1}-\left(n_{1}+n_{2}\right)\right]\left[2\lambda_{s}-\left(n_{1}+n_{2}\right)\right]\left[\lambda_{s}+\lambda_{2}-\left(n_{1}+n_{2}\right)\right]}\times \\ & [\lambda_{s}\left[2\lambda_{s}+\lambda_{1}-\left(n_{1}+n_{2}\right)\right]\left[2\lambda_{s}-\left(n_{1}+n_{2}\right)\right]\left[\lambda_{s}+\lambda_{2}-\left(n_{1}+n_{2}\right)\right]{\bar{v}}_{\left\{3\right\}}^{\left(n_{1}\right)}+\lambda_{s}\lambda_{2}\left[2\lambda_{s}+\lambda_{1}-\left(n_{1}+n_{2}\right)\right]\\ & \times \left[\lambda_{s}+\lambda_{1}+\lambda_{2}-\left(n_{1}+n_{2}\right)\right]{\bar{v}}_{\left\{1\right\}}^{\left(n_{2}\right)}+\lambda_{s}\lambda_{2}\left[\lambda_{s}+\lambda_{2}-\left(n_{1}+n_{2}\right)\right]\left[2\lambda_{s}-\left(n_{1}+n_{2}\right)\right]{\bar{v}}_{\left\{2,3\right\}}^{\left(n_{1}\right)}+\lambda_{s}\lambda_{1}\lambda_{2}\\ & \times \left[\lambda_{s}+\lambda_{2}-\left(n_{1}+n_{2}\right)\right]+\lambda_{s}\lambda_{1}\left[2\lambda_{s}+\lambda_{1}-\left(n_{1}+n_{2}\right)\right]\left[2\lambda_{s}+\lambda_{2}-\left(n_{1}+n_{2}\right)\right]]. \end{array}$$

The final expression of ${\bar{v}}_{\left\{3\},\{1\right\}}^{\left(n_{1},n_{2}\right)}$ in (22) can be obtained by substituting ${\bar{v}}_{\left\{3\right\}}^{\left(n_{1}\right)}$, ${\bar{v}}_{\left\{1\right\}}^{\left(n_{2}\right)}$ and ${\bar{v}}_{\left\{2,3\right\}}^{\left(n_{1}\right)}$ from (21), (A32) and (A30), respectively, into (A37).

## Appendix F. Proof of Proposition 2

The expressions in (27)–(30) of the first moment, second moment, and variance of the age process at each node i∈N={1,2,3} can be derived by plugging the marginal MGF expressions in Theorem 2 into (A22). Furthermore, by plugging the joint MGF ${\bar{v}}_{\left\{3\},\{1\right\}}^{\left(n_{1},n_{2}\right)}$ in (22) into (A23), one can obtain ${\bar{v}}_{\left\{3\},\{1\right\}}^{\left(1,1\right)}$ as follows:

(A38) $$\begin{array}{cc} \;{\bar{v}}_{\left\{3\},\{1\right\}}^{\left(1,1\right)}= & \frac{1}{4\lambda_{s}^{2}\left(\lambda_{s}+\lambda_{1}+\lambda_{2}\right){\left(\lambda_{s}+\lambda_{2}\right)}^{2}\left(2\lambda_{s}+\lambda_{1}\right)\left(\lambda_{1}+\lambda_{2}\right)\left(\lambda_{s}+\lambda_{1}\right)}\times [8\lambda_{s}^{6}+4\lambda_{s}^{5}\left(7\lambda_{1}+8\lambda_{2}\right)+4\lambda_{s}^{4}\left(11\lambda_{1}^{2}+24\lambda_{1}\lambda_{2}+12\lambda_{2}^{2}\right)\\ & +\lambda_{s}^{3}\left(\lambda_{1}+\lambda_{2}\right)\left(32\lambda_{1}^{2}+75\lambda_{1}\lambda_{2}+32\lambda_{2}^{2}\right)+4\lambda_{s}^{2}{\left(\lambda_{1}+\lambda_{2}\right)}^{2}\left(2\lambda_{1}^{2}+9\lambda_{1}\lambda_{2}+2\lambda_{2}^{2}\right)+3\lambda_{s}\lambda_{1}\lambda_{2}\left(3\lambda_{1}^{2}+7\lambda_{1}\lambda_{2}+3\lambda_{2}^{2}\right)\\ & \times \left(\lambda_{1}+\lambda_{2}\right)+3\lambda_{1}^{2}\lambda_{2}^{2}{\left(\lambda_{1}+\lambda_{2}\right)}^{2}], \end{array}$$

The final expression of $\mathrm{cor}\left[x_{1}\left(t\right),x_{3}\left(t\right)\right]$ in (31) can be obtained from (A25) while noting that we have

$$\begin{array}{cc} {\bar{v}}_{\left\{3\},\{1\right\}}^{\left(1,1\right)}-{\bar{v}}_{\left\{3\right\}}^{\left(1\right)}{\bar{v}}_{\left\{1\right\}}^{\left(1\right)}= & \frac{\lambda_{1}\left[8\lambda_{s}^{4}+\lambda_{s}^{3}\left(12\lambda_{1}+7\lambda_{2}\right)+2\lambda_{s}^{2}\left(\lambda_{1}+2\lambda_{2}\right)\left(\lambda_{2}+2\lambda_{1}\right)+\lambda_{s}\lambda_{2}\left(3\lambda_{1}^{2}+5\lambda_{1}\lambda_{2}+\lambda_{2}^{2}\right)+\lambda_{1}\lambda_{2}^{2}\left(\lambda_{1}+\lambda_{2}\right)\right]}{4\lambda_{s}^{2}\left(\lambda_{s}+\lambda_{1}+\lambda_{2}\right){\left(\lambda_{s}+\lambda_{2}\right)}^{2}\left(2\lambda_{s}+\lambda_{1}\right)\left(\lambda_{s}+\lambda_{1}\right)}, \end{array}$$

This completes the proof.

## Appendix G. Proof of Theorem 5

Repeated application of (9) gives

(A39) $$\begin{array}{c} {\bar{v}}_{\left\{1\right\}}^{\left(n\right)}=\frac{\lambda_{c}+\lambda {\bar{v}}_{\left\{1,3\right\}}^{\left(n\right)}}{\lambda_{c}+\lambda -n}, \end{array}$$

(A40) $$\begin{array}{c} {\bar{v}}_{\left\{1,3\right\}}^{\left(n\right)}=\frac{\lambda_{c}+\lambda {\bar{v}}_{\left\{1,2,3\right\}}^{\left(n\right)}}{\lambda_{c}+\lambda -n}\overset{\left(a\right)}{=}\frac{\lambda_{c}}{\lambda_{c}-n}, \end{array}$$

(A41) $$\begin{array}{c} {\bar{v}}_{\left\{1,2,3\right\}}^{\left(n\right)}=\frac{\lambda_{c}}{\lambda_{c}-n}, \end{array}$$

(A42) $$\begin{array}{c} {\bar{v}}_{\left\{2\right\}}^{\left(n\right)}=\frac{\lambda {\bar{v}}_{\left\{1,2\right\}}^{\left(n\right)}}{\lambda -n}, \end{array}$$

(A43) $$\begin{array}{c} {\bar{v}}_{\left\{1,2\right\}}^{\left(n\right)}=\frac{\lambda_{c}+\lambda {\bar{v}}_{\left\{1,2,3\right\}}^{\left(n\right)}}{\lambda_{c}+\lambda -n}\overset{\left(a\right)}{=}\frac{\lambda_{c}}{\lambda_{c}-n}, \end{array}$$

(A44) $$\begin{array}{c} {\bar{v}}_{\left\{3\right\}}^{\left(n\right)}=\frac{\lambda {\bar{v}}_{\left\{2,3\right\}}^{\left(n\right)}}{\lambda -n}, \end{array}$$

(A45) $$\begin{array}{c} {\bar{v}}_{\left\{2,3\right\}}^{\left(n\right)}=\frac{\lambda {\bar{v}}_{\left\{1,2,3\right\}}^{\left(n\right)}}{\lambda -n}\overset{\left(a\right)}{=}\frac{\lambda_{c}\lambda }{\left(\lambda_{c}-n\right)\left(\lambda -n\right)}, \end{array}$$

where step (a) in (A40), (A43), and (A45) follows from substituting ${\bar{v}}_{\left\{1,2,3\right\}}^{\left(n\right)}$ from (A41). The expressions of ${\left\{{\bar{v}}_{\left\{i\right\}}^{\left(n\right)}\right\}}_{i\in \{1,2,3\}}$ in (35)–(37) are obtained from substituting (1) ${\bar{v}}_{\left\{1,3\right\}}^{\left(n\right)}$ from (A40) into (A39), (2) ${\bar{v}}_{\left\{1,2\right\}}^{\left(n\right)}$ from (A43) into (A42), and (3) ${\bar{v}}_{\left\{2,3\right\}}^{\left(n\right)}$ from (A45) into (A44).

Regarding the evaluation of the stationary joint MGF expressions, we start by deriving ${\bar{v}}_{\left\{1\},\{2\right\}}^{\left(n_{1},n_{2}\right)}$. Repeated application of (11) gives

(A46) $$\begin{array}{c} {\bar{v}}_{\left\{1\},\{2\right\}}^{\left(n_{1},n_{2}\right)}\left[\lambda_{c}+2\lambda -\left(n_{1}+n_{2}\right)\right]=\lambda_{c}{\bar{v}}_{\left\{2\right\}}^{\left(n_{2}\right)}+\lambda {\bar{v}}_{\left\{1,3\},\{2\right\}}^{\left(n_{1},n_{2}\right)}+\lambda {\bar{v}}_{\left\{1\},\{1,2\right\}}^{\left(n_{1},n_{2}\right)}, \end{array}$$

(A47) $$\begin{array}{c} {\bar{v}}_{\left\{1,3\},\{2\right\}}^{\left(n_{1},n_{2}\right)}\left[\lambda_{c}+2\lambda -\left(n_{1}+n_{2}\right)\right]=\lambda_{c}{\bar{v}}_{\left\{2\right\}}^{\left(n_{2}\right)}+\lambda {\bar{v}}_{\left\{1,2,3\},\{2\right\}}^{\left(n_{1},n_{2}\right)}+\lambda {\bar{v}}_{\left\{1,3\},\{1,2\right\}}^{\left(n_{1},n_{2}\right)}, \end{array}$$

(A48) $$\begin{array}{c} {\bar{v}}_{\left\{1\},\{1,2\right\}}^{\left(n_{1},n_{2}\right)}\left[\lambda_{c}+\lambda -\left(n_{1}+n_{2}\right)\right]=\lambda_{c}+\lambda {\bar{v}}_{\left\{1,3\},\{1,2\right\}}^{\left(n_{1},n_{2}\right)}, \end{array}$$

(A49) $$\begin{array}{c} {\bar{v}}_{\left\{1,2,3\},\{2\right\}}^{\left(n_{1},n_{2}\right)}\left[\lambda_{c}+\lambda -\left(n_{1}+n_{2}\right)\right]=\lambda_{c}{\bar{v}}_{\left\{2\right\}}^{\left(n_{2}\right)}+\lambda {\bar{v}}_{\left\{1,2,3\},\{1,2\right\}}^{\left(n_{1},n_{2}\right)}, \end{array}$$

(A50) $$\begin{array}{c} {\bar{v}}_{\left\{1,3\},\{1,2\right\}}^{\left(n_{1},n_{2}\right)}\left[\lambda_{c}+2\lambda -\left(n_{1}+n_{2}\right)\right]=\lambda_{c}+\lambda {\bar{v}}_{\left\{1,2,3\},\{1,2\right\}}^{\left(n_{1},n_{2}\right)}+\lambda {\bar{v}}_{\left\{1,3\},\{1,2,3\right\}}^{\left(n_{1},n_{2}\right)}, \end{array}$$

(A51) $$\begin{array}{c} {\bar{v}}_{\left\{1,2,3\},\{1,2\right\}}^{\left(n_{1},n_{2}\right)}\left[\lambda_{c}+\lambda -\left(n_{1}+n_{2}\right)\right]=\lambda_{c}+\lambda {\bar{v}}_{\left\{1,2,3\},\{1,2,3\right\}}^{\left(n_{1},n_{2}\right)}, \end{array}$$

(A52) $$\begin{array}{c} {\bar{v}}_{\left\{1,3\},\{1,2,3\right\}}^{\left(n_{1},n_{2}\right)}\left[\lambda_{c}+\lambda -\left(n_{1}+n_{2}\right)\right]=\lambda_{c}+\lambda {\bar{v}}_{\left\{1,2,3\},\{1,2,3\right\}}^{\left(n_{1},n_{2}\right)}, \end{array}$$

(A53) $$\begin{array}{c} {\bar{v}}_{\left\{1,2,3\},\{1,2,3\right\}}^{\left(n_{1},n_{2}\right)}=\frac{\lambda_{c}}{\lambda_{c}-\left(n_{1}+n_{2}\right)}. \end{array}$$

By substituting ${\bar{v}}_{\left\{1,2,3\},\{1,2,3\right\}}^{\left(n_{1},n_{2}\right)}$ from (A53) into (A48) and (A50)–(A52), we obtain

(A54) $$\begin{array}{c} {\bar{v}}_{\left\{1,2,3\},\{1,2\right\}}^{\left(n_{1},n_{2}\right)}={\bar{v}}_{\left\{1,3\},\{1,2,3\right\}}^{\left(n_{1},n_{2}\right)}={\bar{v}}_{\left\{1,3\},\{1,2\right\}}^{\left(n_{1},n_{2}\right)}={\bar{v}}_{\left\{1\},\{1,2\right\}}^{\left(n_{1},n_{2}\right)}=\frac{\lambda_{c}}{\lambda_{c}-\left(n_{1}+n_{2}\right)}, \end{array}$$

Furthermore, from (A47), (A49), (A50) and (A54), ${\bar{v}}_{\left\{1,3\},\{2\right\}}^{\left(n_{1}.n_{2}\right)}$ can be expressed as

(A55) $$\begin{array}{c} {\bar{v}}_{\left\{1,3\},\{2\right\}}^{\left(n_{1},n_{2}\right)}=\frac{\lambda_{c}\left[\lambda_{c}-\left(n_{1}+n_{2}\right)\right]{\bar{v}}_{\left\{2\right\}}^{\left(n_{2}\right)}+\lambda \lambda_{c}}{\left[\lambda_{c}+\lambda -\left(n_{1}+n_{2}\right)\right]\left[\lambda_{c}-\left(n_{1}+n_{2}\right)\right]}. \end{array}$$

The final expression of ${\bar{v}}_{\left\{1\},\{2\right\}}^{\left(n_{1},n_{2}\right)}$ in (38) can be obtained by substituting ${\bar{v}}_{\left\{1,3\},\{2\right\}}^{\left(n_{1},n_{2}\right)}$, ${\bar{v}}_{\left\{1\},\{1,2\right\}}^{\left(n_{1},n_{2}\right)}$, and ${\bar{v}}_{\left\{2\right\}}^{\left(n_{2}\right)}$ from (A55), (A54) and (36), respectively, into (A46). Now, we proceed with the evaluation of ${\bar{v}}_{\left\{1\},\{3\right\}}^{\left(n_{1},n_{2}\right)}$. Repeated application of (11) gives

(A56) $$\begin{array}{c} {\bar{v}}_{\left\{1\},\{3\right\}}^{\left(n_{1},n_{2}\right)}\left[\lambda_{c}+2\lambda -\left(n_{1}+n_{2}\right)\right]=\lambda_{c}{\bar{v}}_{\left\{3\right\}}^{\left(n_{2}\right)}+\lambda {\bar{v}}_{\left\{1,3\},\{3\right\}}^{\left(n_{1},n_{2}\right)}+\lambda {\bar{v}}_{\left\{1\},\{2,3\right\}}^{\left(n_{1},n_{2}\right)}, \end{array}$$

(A57) $$\begin{array}{c} {\bar{v}}_{\left\{1,3\},\{3\right\}}^{\left(n_{1},n_{2}\right)}\left[\lambda_{c}+\lambda -\left(n_{1}+n_{2}\right)\right]=\lambda_{c}{\bar{v}}_{\left\{3\right\}}^{\left(n_{2}\right)}+\lambda {\bar{v}}_{\left\{1,2,3\},\{2,3\right\}}^{\left(n_{1},n_{2}\right)}, \end{array}$$

(A58) $$\begin{array}{c} {\bar{v}}_{\left\{1\},\{2,3\right\}}^{\left(n_{1},n_{2}\right)}\left[\lambda_{c}+2\lambda -\left(n_{1}+n_{2}\right)\right]=\lambda_{c}{\bar{v}}_{\left\{2,3\right\}}^{\left(n_{2}\right)}+\lambda {\bar{v}}_{\left\{1,3\},\{2,3\right\}}^{\left(n_{1},n_{2}\right)}+\lambda {\bar{v}}_{\left\{1\},\{1,2,3\right\}}^{\left(n_{1},n_{2}\right)}, \end{array}$$

(A59) $$\begin{array}{c} {\bar{v}}_{\left\{1,2,3\},\{2,3\right\}}^{\left(n_{1},n_{2}\right)}\left[\lambda_{c}+\lambda -\left(n_{1}+n_{2}\right)\right]=\lambda_{c}{\bar{v}}_{\left\{2,3\right\}}^{\left(n_{2}\right)}+\lambda {\bar{v}}_{\left\{1,2,3\},\{1,2,3\right\}}^{\left(n_{1},n_{2}\right)}, \end{array}$$

(A60) $$\begin{array}{c} {\bar{v}}_{\left\{1,3\},\{2,3\right\}}^{\left(n_{1},n_{2}\right)}\left[\lambda_{c}+2\lambda -\left(n_{1}+n_{2}\right)\right]=\lambda_{c}{\bar{v}}_{\left\{2,3\right\}}^{\left(n_{2}\right)}+\lambda {\bar{v}}_{\left\{1,3\},\{1,2,3\right\}}^{\left(n_{1},n_{2}\right)}+\lambda {\bar{v}}_{\left\{1,2,3\},\{2,3\right\}}^{\left(n_{1},n_{2}\right)}, \end{array}$$

(A61) $$\begin{array}{c} {\bar{v}}_{\left\{1\},\{1,2,3\right\}}^{\left(n_{1},n_{2}\right)}\left[\lambda_{c}+\lambda -\left(n_{1}+n_{2}\right)\right]=\lambda_{c}+\lambda {\bar{v}}_{\left\{1,3\},\{1,2,3\right\}}^{\left(n_{1},n_{2}\right)}, \end{array}$$

where ${\bar{v}}_{\left\{1,3\},\{1,2,3\right\}}^{\left(n_{1},n_{2}\right)}={\bar{v}}_{\left\{1,2,3\},\{1,2,3\right\}}^{\left(n_{1},n_{2}\right)}=\frac{\lambda_{c}}{\lambda_{c}-\left(n_{1}+n_{2}\right)}$. By substituting ${\bar{v}}_{\left\{1,3\},\{1,2,3\right\}}^{\left(n_{1},n_{2}\right)}$ into (A61), we obtain

(A62) $$\begin{array}{c} {\bar{v}}_{\left\{1\},\{1,2,3\right\}}^{\left(n_{1},n_{2}\right)}=\frac{\lambda_{c}}{\lambda_{c}-\left(n_{1}+n_{2}\right)}. \end{array}$$

Furthermore, from (A57)–(A62), ${\bar{v}}_{\left\{1,3\},\{3\right\}}^{\left(n_{1},n_{2}\right)}$ and ${\bar{v}}_{\left\{1\},\{2,3\right\}}^{\left(n_{1},n_{2}\right)}$ can be respectively expressed as

(A63) $$\begin{array}{c} {\bar{v}}_{\left\{1,3\},\{3\right\}}^{\left(n_{1},n_{2}\right)}=\frac{\lambda_{c}\left[\lambda_{c}+\lambda -\left(n_{1}+n_{2}\right)\right]{\bar{v}}_{\left\{3\right\}}^{\left(n_{2}\right)}+\lambda \left(\lambda_{c}{\bar{v}}_{\left\{2,3\right\}}^{\left(n_{2}\right)}+\lambda {\bar{v}}_{\left\{1,2,3\},\{1,2,3\right\}}^{\left(n_{1},n_{2}\right)}\right)}{{\left[\lambda_{c}+\lambda -\left(n_{1}+n_{2}\right)\right]}^{2}}, \end{array}$$

(A64) $$\begin{array}{c} {\bar{v}}_{\left\{1\},\{2,3\right\}}^{\left(n_{1},n_{2}\right)}=\frac{\lambda_{c}{\bar{v}}_{\left\{2,3\right\}}^{\left(n_{2}\right)}+\lambda {\bar{v}}_{\left\{1\},\{1,2,3\right\}}^{\left(n_{1},n_{2}\right)}}{\lambda_{c}+\lambda -\left(n_{1}+n_{2}\right)}. \end{array}$$

The final expression of ${\bar{v}}_{\left\{1\},\{3\right\}}^{\left(n_{1},n_{2}\right)}$ in (39) can be obtained from substituting (A63) and (A64) into (A56), followed by some algebraic simplifications. Finally, to derive ${\bar{v}}_{\left\{2\},\{3\right\}}^{\left(n_{1},n_{2}\right)}$, we first repeatedly use (11) as follows:

(A65) $$\begin{array}{c} {\bar{v}}_{\left\{2\},\{3\right\}}^{\left(n_{1},n_{2}\right)}\left[2\lambda -\left(n_{1}+n_{2}\right)\right]=\lambda {\bar{v}}_{\left\{1,2\},\{3\right\}}^{\left(n_{1},n_{2}\right)}+\lambda {\bar{v}}_{\left\{2\},\{2,3\right\}}^{\left(n_{1},n_{2}\right)}, \end{array}$$

(A66) $$\begin{array}{c} {\bar{v}}_{\left\{1,2\},\{3\right\}}^{\left(n_{1},n_{2}\right)}\left[\lambda_{c}+2\lambda -\left(n_{1}+n_{2}\right)\right]=\lambda_{c}{\bar{v}}_{\left\{3\right\}}^{\left(n_{2}\right)}+\lambda {\bar{v}}_{\left\{1,2,3\},\{3\right\}}^{\left(n_{1},n_{2}\right)}+\lambda {\bar{v}}_{\left\{1,2\},\{2,3\right\}}^{\left(n_{1},n_{2}\right)}, \end{array}$$

(A67) $$\begin{array}{c} {\bar{v}}_{\left\{2\},\{2,3\right\}}^{\left(n_{1},n_{2}\right)}\left[\lambda -\left(n_{1}+n_{2}\right)\right]=\lambda {\bar{v}}_{\left\{1,2\},\{1,2,3\right\}}^{\left(n_{1},n_{2}\right)}, \end{array}$$

(A68) $$\begin{array}{c} {\bar{v}}_{\left\{1,2,3\},\{3\right\}}^{\left(n_{1},n_{2}\right)}\left[\lambda_{c}+\lambda -\left(n_{1}+n_{2}\right)\right]=\lambda_{c}{\bar{v}}_{\left\{3\right\}}^{\left(n_{2}\right)}+\lambda {\bar{v}}_{\left\{1,2,3\},\{2,3\right\}}^{\left(n_{1},n_{2}\right)}, \end{array}$$

(A69) $$\begin{array}{c} {\bar{v}}_{\left\{1,2\},\{2,3\right\}}^{\left(n_{1},n_{2}\right)}\left[\lambda_{c}+2\lambda -\left(n_{1}+n_{2}\right)\right]=\lambda_{c}{\bar{v}}_{\left\{2,3\right\}}^{\left(n_{2}\right)}+\lambda {\bar{v}}_{\left\{1,2,3\},\{2,3\right\}}^{\left(n_{1},n_{2}\right)}+\lambda {\bar{v}}_{\left\{1,2\},\{1,2,3\right\}}^{\left(n_{1},n_{2}\right)}. \end{array}$$

From (A66)–(A69), ${\bar{v}}_{\left\{1,2\},\{3\right\}}^{\left(n_{1},n_{2}\right)}$ and ${\bar{v}}_{\left\{2\},\{2,3\right\}}^{\left(n_{1},n_{2}\right)}$ can be respectively expressed as

(A70) $$\begin{array}{cc} {\bar{v}}_{\left\{1,2\},\{3\right\}}^{\left(n_{1},n_{2}\right)}= & \frac{1}{\left[\lambda_{c}+\lambda -\left(n_{1}+n_{2}\right)\right]{\left[\lambda_{c}+2\lambda -\left(n_{1}+n_{2}\right)\right]}^{2}}\times [\lambda_{c}{\left[\lambda_{c}+2\lambda -\left(n_{1}+n_{2}\right)\right]}^{2}{\bar{v}}_{\left\{3\right\}}^{\left(n_{2}\right)}\\ & +\lambda \left[\lambda_{c}+\lambda -\left(n_{1}+n_{2}\right)\right]\left(\lambda_{c}{\bar{v}}_{\left\{2,3\right\}}^{\left(n_{2}\right)}+\lambda {\bar{v}}_{\left\{1,2\},\{1,2,3\right\}}^{\left(n_{1},n_{2}\right)}\right)+\lambda^{2}\left[2\lambda_{c}+3\lambda -2\left(n_{1}+n_{2}\right)\right]{\bar{v}}_{\left\{1,2,3\},\{2,3\right\}}^{\left(n_{1},n_{2}\right)}], \end{array}$$

(A71) $$\begin{array}{c} {\bar{v}}_{\left\{2\},\{2,3\right\}}^{\left(n_{1},n_{2}\right)}=\frac{\lambda_{c}\lambda }{\left[\lambda_{c}-\left(n_{1}+n_{2}\right)\right]\left[\lambda -\left(n_{1}+n_{2}\right)\right]}. \end{array}$$

The final expression of ${\bar{v}}_{\left\{2\},\{3\right\}}^{\left(n_{1},n_{2}\right)}$ in (40) can be obtained from plugging (A70) and (A71) into (A65), followed by substituting (1) ${\bar{v}}_{\left\{3\right\}}^{\left(n_{2}\right)}$ from (37), (2) ${\bar{v}}_{\left\{2,3\right\}}^{\left(n_{2}\right)}$ from (A45), (3) ${\bar{v}}_{\left\{1,2,3\},\{2,3\right\}}^{\left(n_{1},n_{2}\right)}$ from (A59), and (4) ${\bar{v}}_{\left\{1,2\},\{1,2,3\right\}}^{\left(n_{1},n_{2}\right)}$ as $\frac{\lambda_{c}}{\lambda_{c}-\left(n_{1}+n_{2}\right)}$.

## Appendix H. Proof of Proposition 3

The results of this proposition can be derived by following similar steps to those in Appendix D and Appendix F while noting that

(A72) $$\begin{array}{c} {\bar{v}}_{\left\{1\},\{2\right\}}^{\left(1,1\right)}=\frac{\lambda^{2}+{\left(\lambda_{c}+\lambda \right)}^{2}}{\lambda \lambda_{c}^{2}\left(\lambda_{c}+\lambda \right)}, \end{array}$$

(A73) $$\begin{array}{c} {\bar{v}}_{\left\{1\},\{2\right\}}^{\left(1,1\right)}-{\bar{v}}_{\left\{1\right\}}^{\left(1\right)}{\bar{v}}_{\left\{2\right\}}^{\left(1\right)}=\frac{\lambda }{\lambda_{c}^{2}\left(\lambda_{c}+\lambda \right)}, \end{array}$$

(A74) $$\begin{array}{c} {\bar{v}}_{\left\{1\},\{3\right\}}^{\left(1,1\right)}=\frac{2\lambda_{c}^{3}+5\lambda_{c}^{2}\lambda +4\lambda_{c}\lambda^{2}+2\lambda^{3}}{\lambda \lambda_{c}^{2}{\left(\lambda_{c}+\lambda \right)}^{2}}, \end{array}$$

(A75) $$\begin{array}{c} {\bar{v}}_{\left\{1\},\{3\right\}}^{\left(1,1\right)}-{\bar{v}}_{\left\{1\right\}}^{\left(1\right)}{\bar{v}}_{\left\{3\right\}}^{\left(1\right)}=\frac{\lambda^{2}}{\lambda_{c}^{2}{\left(\lambda_{c}+\lambda \right)}^{2}}, \end{array}$$

(A76) $$\begin{array}{c} {\bar{v}}_{\left\{2\},\{3\right\}}^{\left(1,1\right)}=\frac{5\lambda_{c}^{4}+16\lambda_{c}^{3}\lambda +20\lambda_{c}^{2}\lambda^{2}+12\lambda_{c}\lambda^{3}+4\lambda^{4}}{2\lambda_{c}^{2}\lambda^{2}{\left(\lambda_{c}+\lambda \right)}^{2}}, \end{array}$$

(A77) $$\begin{array}{c} {\bar{v}}_{\left\{2\},\{3\right\}}^{\left(1,1\right)}-{\bar{v}}_{\left\{2\right\}}^{\left(1\right)}{\bar{v}}_{\left\{3\right\}}^{\left(1\right)}=\frac{\lambda_{c}^{4}+2\lambda_{c}^{3}\lambda +2\lambda_{c}^{2}\lambda^{2}+2\lambda_{c}\lambda^{3}+2\lambda^{4}}{2\lambda_{c}^{2}\lambda^{2}{\left(\lambda_{c}+\lambda \right)}^{2}}. \end{array}$$

## Appendix I. Proof of Proposition 4

We first apply (11) to obtain ${\bar{v}}_{N_{i},N_{j}}^{\left(n_{1},n_{2}\right)}$ as

(A78) $$\begin{array}{c} {\bar{v}}_{N_{i},N_{j}}^{\left(n_{1},n_{2}\right)}=\frac{\lambda_{i}{\bar{v}}_{N_{j}}^{\left(n_{2}\right)}+\lambda_{j}{\bar{v}}_{N_{i}}^{\left(n_{1}\right)}}{\lambda_{i}+\lambda_{j}-\left(n_{1}+n_{2}\right)}\overset{\left(a\right)}{=}\frac{\lambda_{i}\lambda_{j}}{\left(\lambda_{i}-n_{1}\right)\left(\lambda_{j}-n_{2}\right)}, \end{array}$$

where step (a) follows from substituting ${\bar{v}}_{N_{i}}^{\left(n_{1}\right)}$ and ${\bar{v}}_{N_{j}}^{\left(n_{2}\right)}$ from (A41) as $\frac{\lambda_{i}}{\lambda_{i}-n_{1}}$ and $\frac{\lambda_{j}}{\lambda_{j}-n_{2}}$, respectively. We then obtain $\frac{\partial^{2}{\bar{v}}_{N_{i},N_{j}}^{\left(n_{1},n_{2}\right)}}{\partial n_{2}\partial n_{1}}$ as

(A79) $$\begin{array}{c} \frac{\partial^{2}{\bar{v}}_{N_{i},N_{j}}^{\left(n_{1},n_{2}\right)}}{\partial n_{2}\partial n_{1}}=\frac{\lambda_{i}\lambda_{j}}{{\left(\lambda_{i}-n_{1}\right)}^{2}{\left(\lambda_{j}-n_{2}\right)}^{2}}. \end{array}$$

Thus, from (A79), we have

(A80) $$\begin{array}{c} {\bar{v}}_{N_{i},N_{j}}^{\left(1,1\right)}=\frac{1}{\lambda_{i}\lambda_{j}}. \end{array}$$

The conclusion that the two age processes $x_{N_{i}}\left(t\right)$ and $x_{N_{j}}\left(t\right)$ are uncorrelated follows from noting that ${\bar{v}}_{N_{i},N_{j}}^{\left(1,1\right)}-{\bar{v}}_{N_{i}}^{\left(1\right)}{\bar{v}}_{N_{j}}^{\left(1\right)}=0$, and hence the correlation coefficient between $x_{N_{i}}\left(t\right)$ and $x_{N_{j}}\left(t\right)$ is zero.
